# Integrated analyses of ionomics, phytohormone profiles, transcriptomics, and metabolomics reveal a pivotal role of carbon-nano sol in promoting the growth of tobacco plants

**DOI:** 10.1186/s12870-024-05195-1

**Published:** 2024-05-30

**Authors:** Chen Wang, Yingpeng Hua, Taibo Liang, Yadi Guo, Lin Wang, Xueao Zheng, Pingping Liu, Qingxia Zheng, Zhengzhong Kang, Yalong Xu, Peijian Cao, Qiansi Chen

**Affiliations:** 1 Zhengzhou Tobacco Research Institute of CNTC, Zhengzhou, 450001 China; 2 School of Agricultural Sciences, Zhengzhou University, Zhengzhou, 450001 China; 3 Beijing Life Science Academy (BLSA), Beijing, 102209 China

**Keywords:** Auxin, Carbon-nano sol, Nitrate allocation, Potassium, Tobacco

## Abstract

**Background:**

Carbon nano sol (CNS) can markedly affect the plant growth and development. However, few systematic analyses have been conducted on the underlying regulatory mechanisms in plants, including tobacco (*Nicotiana tabacum* L.).

**Results:**

Integrated analyses of phenome, ionome, transcriptome, and metabolome were performed in this study to elucidate the physiological and molecular mechanisms underlying the CNS-promoting growth of tobacco plants. We found that 0.3% CNS, facilitating the shoot and root growth of tobacco plants, significantly increased shoot potassium concentrations. Antioxidant, metabolite, and phytohormone profiles showed that 0.3% CNS obviously reduced reactive oxygen species production and increased antioxidant enzyme activity and auxin accumulation. Comparative transcriptomics revealed that the GO and KEGG terms involving responses to oxidative stress, DNA binding, and photosynthesis were highly enriched in response to exogenous CNS application. Differential expression profiling showed that *NtNPF7.3/NtNRT1.5*, potentially involved in potassium/auxin transport, was significantly upregulated under the 0.3% CNS treatment. High-resolution metabolic fingerprints showed that 141 and 163 metabolites, some of which were proposed as growth regulators, were differentially accumulated in the roots and shoots under the 0.3% CNS treatment, respectively.

**Conclusions:**

Taken together, this study revealed the physiological and molecular mechanism underlying CNS-mediated growth promotion in tobacco plants, and these findings provide potential support for improving plant growth through the use of CNS.

**Supplementary Information:**

The online version contains supplementary material available at 10.1186/s12870-024-05195-1.

## Background

Nanomaterials in agriculture offers innovative possibilities for sustainable farming practices [1]. In the field of plant biology, extensive investigations have revealed that nanomaterials possess the capability to be absorbed by plants, transported to different tissues and organs, and exert a positive influence on plant growth, development [2], and seed quality [3]. Carbon nanomaterials, including carbon nanotubes, graphene, fullerenes, carbon nano-horns, carbon-nano sol (CNS), and carbon nano-particles, have piqued the interest of researchers because of their unusual chemical characteristics, structures, and low biotoxicity [2]. The agricultural applications of carbon nanomaterials, particularly their influence on plant growth and development, have garnered increasing attention from researchers seeking innovative approaches to enhance agricultural sustainability [4].

Studies exploring the physiological bases and mechanisms underlying the plant-promoting effects of carbon nanomaterials have unveiled intriguing outcomes. For instance, carbon nanotubes have been shown to impact root elongation in diverse crop species, such as cucumber, onion, lettuce, and tomato [2]. In wheat, water-soluble carbon nanoparticles were demonstrated to regulate nutrient release, facilitating enhanced assimilation by plants and consequently promoting growth [5]. Single-walled carbon nano-horns were found to activate seed germination in specific crops and stimulate the growth of various organs in corn, tomato, rice, and soybean [6].

Moreover, the influence of carbon nanotubes on maize revealed increased activities of the enzymes associated with nitrogen assimilation (such as glutamate synthetase and glutamine oxoglutarate aminotransferase), along with elevated pyruvate content in shoots and roots, promoting carbohydrate synthesis and nitrogen use efficiency [7]. In the context of tobacco, carbon-based nanomaterials were observed to enhance plant immunity by improving photosynthetic performance and eliciting defense responses against tobacco mosaic virus [8]. In addition, multi-walled carbon nanotubes exhibited the capacity to augment the tobacco cell growth [9]. CNS, a distinctive form of carbon nanomaterials with average sizes of 30 μm, is generated through the electrolysis of graphite using the pulsed electrode method, offering advantages, such as biological properties, excellent compatibility, facile preparation, and cost-effectiveness [10]. CNS is proposed to enter plant cells mainly through endocytosis [11].

Extensive studies have demonstrated the capacity of carbon nanomaterials to enhance crop production [12], stimulate root elongation [13], facilitate nutrient element accumulation, and modulate overall plant growth and development. Specifically, CNS exhibits great promise in improving crop fertilizer utilization, augmenting root vitality, and enhancing crop quality [7–9].

Tobacco (*Nicotiana tabacum* L.), an allopolyploid species (2n = 4x = 48), originated from a tetraploidization event with a basic chromosome number (*n* = 12), sharing this genomic characteristic with several solanaceous species such as tomato, potato, pepper, and eggplant [14]. Tobacco is renowned as one of the most widely cultivated non-food cash crops and a model organism for plant molecular and metabolic studies [15]. Nanomaterials have been reported to boost the growth of tobacco [16]. Notably, zinc oxide nanoparticles have been shown to alleviate tobacco growth under cadmium toxicity by reprogramming key metabolic pathways, including alkaloids, amino acids, and flavonoids [17]. In addition, carbon nanomaterials have been reported to enhance potassium absorption by upregulating the expression of potassium channels, mimicking biological ion channels in tobacco BY-2 cells [18]. However, the underlying physiological and molecular mechanisms by which CNS regulates the growth and development of tobacco remain unknown as yet.

In the present study, we conducted a comprehensive analysis of the phenome, ionome, transcriptome, and metabolome in tobacco, aimed to dissect the mechanisms involved in the promotion of tobacco plant growth by CNS. In addition, our findings hold the potential to contribute valuable insights for the application of CNS in agricultural production, thereby advancing sustainable farming practices and enhancing crop yields.

## Methods

### Materials and growth conditions

Tobacco (*Nicotiana tobacum* L., cv. K326) seeds, and CNS were provided by the Zhengzhou Tobacco Research Institute of the China National Tobacco Corporation. The preparation details of CNS have been in detail described by Cheng et al. [19].

Seeds of uniform sizes were selected and sterilized with 1% NaClO for 10 min, which was followed by rinse and soak in pure water for 24 h at 4ºC. Uniform tobacco seedlings were hydroponically grown in an illuminated growth chamber using the full-strength Hoagland and Arnon nutrient solution. The basic nutrient solution used in this study was as follows: 1.0 mM KH_2_PO_4_, 5.0 mM KNO_3_, 5.0 mM Ca(NO_3_)_2_·4H_2_O, 2.0 mM MgSO_4_·7H_2_O, 50 µM EDTA-Fe(II), 9.0 µM MnCl_2_·4H_2_O, 0.80 µM ZnSO_4_·7H_2_O, 0.30 µM CuSO_4_·5H_2_O, 0.37 µM Na_2_MoO_4_·2H_2_O, and 46 µM H_3_BO_3_. The nutrient solution was refreshed every 5 d until sampling. The room temperature was set at 24ºC, with a photoperiod of 14 h (light)/10 h (dark), light intensity of 300–320 µmol m^− 2^ s^− 1^, and humidity of 60-75%.

### Characterization of CNS and determination of physiological parameters

The ultrastructure of CNS was characterized using a scanning electron microscope/SEM (JSM-6390/LV, JEOL, Tokyo, Japan). Each test included at least ten independent biological replicates. The maximum root length of each tobacco plant was measured after 15-d hydroponic culture. The fresh shoots and roots of tobacco seedlings were collected separately and de-enzyming at 105ºC for 30 min, followed by oven-drying at 65ºC until a constant weight was achieved. The roots from the sampled tobacco seedlings were imaged under a scanner (Epson Perfection V800 photo, Epson, Suwa, Japan) to determine total root length, root volume, root surface area, and average root diameter, which were analyzed using WinRHIZO Pro (Regent Instruments, QC, Canada).

For the quantification of proline levels, the procedure involved harvesting plant samples, determining their fresh weight and using approximately 100 mg for the subsequent reaction. The fresh samples were ground after the addition of 3% sulfosalicylic acid (5 µL/mg fresh weight), followed by centrifugation for 5 min. A 100 µL reaction mixture (comprising 3% sulfosalicylic acid, glacial acetic acid, and acidic ninhydrin in a ratio of 1:2:2) was added to the supernatant obtained from the plant extract. Subsequently, the samples were extracted with toluene, and the absorbance was measured at 520 nm, with toluene serving as the reference [20].

For the determination of malondialdehyde (MDA) levels, thiobarbituric acid (TBA) was employed for extraction, and the concentration was further assessed spectrophotometrically at the wavelengths of 450, 532, and 600 nm, respectively [21]. For the determination of superoxide anion (O_2_^−^), a histochemical assay was employed. Initially, the tobacco leaves were excised and placed, abaxial side up, in a staining solution comprising 0.1% (w/v) NBT, 10 mM sodium azide, and 50 mM potassium phosphate at pH 6.4. Subsequently, the leaves were incubated in a 10 mL staining solution (0.1% NBT) for 15 min. Total chlorophyll was then removed through a series of ethanol washes; O_2_^−^ reacts with NBT, resulting in a blue stain [22].

To quantify the hydrogen peroxide (H_2_O_2_) content, a reaction mixture was prepared, consisting of 1 mL of reagent (0.1% v/v TiCl_4_ in 20% v/v H_2_SO_4_) and 2 mL of the 50 mM phosphate-buffered leaf extract supernatant at pH 6.8. The blank process involved the absence of leaf extract in the 50 mM phosphate buffer. Spectrophotometric analysis of the H_2_O_2_ level was conducted following the reaction with TiCl_4_ [23]. For each group sample, five independent biological replicates, each with three technical replicates, were tested under the same conditions.

### Ionomic analysis

After the treatment of tobacco seedlings with 0.3% CNS for 15 d, the shoot and root tissues of fresh tobacco seedlings were collected, oven-dried at 65 °C to constant weight, and weighed for biomass. Subsequently, the samples were digested with HNO_3_:HClO_4_ (v: v, 4:1) at 200 °C until completely dissolved. The diluted supernatant was submitted to ICP-MS (Agilent ICAP 7000, Palo Alto, CA, USA) [24]. For each group sample, five independent biological replicates, each with three technical replicates, were tested under the same conditions.

### Non-invasive micro-tests

The net fluxes of H^+^, NO_3_^−^, and IAA were measured using NMT (NMT100 Series, Younger, USA LLC, Amherst, MA, USA; Xuyue (Beijing) Sci. & Tech. Co., Ltd., Beijing, China) and imFluxes V2.0 (Younger, USA LLC, Amherst, MA, USA) Software. For the IAA assay, sensor construction, surface modification, and calibration were performed using the methods described by McLamore et al. [25]. For the H^+^ and NO_3_^−^ assay, electrodes were fabricated as described by Yan et al. [26]. The maximal net fluxes of H^+^, NO_3_^−^, and IAA along the root tips were determined by measuring at least eight similar roots for each treatment. The recording rate for the ion flux was one reading per six seconds. The net flux data were processed using Mageflux (Younger USA Corp., https://youngerusa.com/mageflux) and based on Fick’s law of diffusion.

### Determination of phytohormone contents

Fresh shoot and root samples from tobacco plants treated with 0.3% CNS for 15 d were ground in liquid nitrogen, then the resultant powder (∼ 0.1 g) was subjected to an ice-cold extraction buffer (methanol: water: acetic acid, 80:19:1, v/v/v) to extract phytohormones [27]. The phytohormone concentrations in the extracts were determined by ultra-fast liquid chromatography-electrospray ionization tandem mass spectrometry (QTrap6500, SCICEX, Boston, MA, USA) [28]. Standards for auxin (indole-3-acetic acid, IAA), cytokinin (trans-zeatin, *t*Z), gibberellin (GA_3_), abscisic acid (ABA), jasmonic acid (JA), and salicylic acid (SA) were obtained from Sigma-Aldrich. For each group sample, five independent biological replicates, each with three technical replicates, were tested under the same conditions.

### RNA-sequencing and transcriptomic analysis

The shoots and roots of the afore-mentioned tobacco plants were harvested, and three independent biological replicates were used for each group sample. Pre-chilled Trizol (Takara Bio Inc, Kusatsu, Shiga, Japan) was used to isolate total RNA. The values of RNA integrity number (> 8.0) of these samples were assessed using an Agilent 2100 Bioanalyzer (Santa Clara, CA, USA). Samples of RNA with the RIN values > 8.0 were obtained to construct strand-specific cDNA libraries. Paired-end transcriptome sequencing was performed on the Illumina HiSeq X Ten sequencing platform (Illumina Hiseq 4000, San Diego, CA, USA), with a single read length of 150 bp. Approximately, 10.0 Gb sequencing data were obtained for each sample. The raw paired-end reads were trimmed and quality controlled by fastp with default parameters. Then, clean reads were separately aligned to the reference genome with orientation mode using the HISAT2 software. The mapped reads of each sample were assembled by StringTie in a reference-based approach. To identify differentially expressed genes (DEGs) between two different samples, the expression level of each transcript was normalized based on the transcripts per million reads (TPM) method. The RSEM (RNA-sequencing by Expectation-Maximization) software was used to quantify gene expression abundances. Differential expression analysis was performed using DESeq2 (https://bioconductor.org/packages/release/bioc/html/DESeq2.html), and the DEGs were defined as those genes with |log_2_(fold change)| ≥ 1 and false discovery rate < 0.05. In addition, functional-enrichment analysis, including functional annotation of gene ontology (GO) and Kyoto Encyclopedia of Genes and Genomes (KEGG), was performed to identify which DEGs were significantly enriched in GO terms and metabolic pathways at the Bonferroni-corrected P-value < 0.05 compared with the whole-transcriptome background. GO functional enrichment and KEGG pathway analysis were carried out using Goatools and Python scipy, respectively.

### Genome-wide identification and molecular characterization of *NPF* family genes in tobacco

Previous studies have suggested that nitrate peptide family (NPF) transporters have multiple transport activity of various substrates, such as nutrients and phytohormones, and are widely involved in the biological processes [29]. The genomic, coding sequences, and protein sequences of *NPFs* in *A. thaliana* and *N. tabacum* were retrieved from the Arabidopsis Information Resource (TAIR10, https://www.arabidopsis.org) and the Sol Genomics Network (https://solgenomics.net/organism/Nicotiana_tabacum/genome). To identify the *NPF* homologs in tobacco, the *NPF* protein sequences from Arabidopsis were used as queries through a reciprocal Basic Local Alignment Search Tool (BLAST) analysis using the threshold and minimum alignment coverage parameters described previously. All the NPF protein sequences were confirmed by comparison with the *NPF* sequences through searches of the NCBI-CDD database (https://www.ncbi.nlm.nih.gov/Structure/bwrpsb/bwrpsb.cgi).

Multiple sequence alignments of the coding sequences of *NPFs* between *A. thaliana* and *N. tabacum* were aligned using ClustalW (https://www.genome.jp/tools-bin/clustalw) with default parameters. Phylogenetic analysis of NPF proteins was performed using Molecular Evolutionary Genetics Analysis (MEGA) 7.0 program with the neighbor-joining (NJ) method based on the p-distance + G substitution model. The bootstrap method was used to test the tree with 1000 replicates, and conserved sequences with a coverage of 70%. The phylogenetic trees were visualized using iTOL (V5, https://itol.embl.de/).

To identify conserved motifs in the proteins, we employed the Expectation Maximization for Motif Elucidation program (MEME v4.12.0, https://meme-suite.org/meme/), utilizing specific parameter settings, including a maximum motif number set at 10. The PFAM domain (PF00854.18: PTR2) associated with the Major Facilitator Superfamily (MFS) of membrane transport proteins was utilized for the identification of *NtNPFs*. The NCBI Conserved Domain Database was used to search for the identification of the conserved domains of NPFs. Visualization of motifs, gene structure, and conserved domains of the candidate genes was achieved using TBtools-II. Physical locations of *NtNPFs* in the *N. tabacum* genome were collected from the Sol Genomics Network database (https://solgenomics.net/organism/Nicotiana_tabacum/genome), and were drafted to chromosomes by using TBtools-II. To uncover the evolutionary linear relationships between *A. thaliana* and *N. tabacum*, the MCScanX plugin in TBtools-II was used to perform collinearity analysis.

The *cis*-acting regulatory elements (CREs) in the promoter regions of target genes are thought to play key roles in the regulation of gene expression. To further investigate the potential regulatory networks of *NtNPFs*, the 2,000 bp upstream genomic DNA sequences of the start codon (ATG) were submitted to PlantCARE (http://bioinformatics.psb.ugent.be/webtools/plantcare/html/) to obtain the potential CREs.

### High-resolution metabolomic fingerprint analysis

After seed germination, uniform seedlings were grown hydroponically under the CNS-free and 0.3% CNS solution conditions for 15 d until sampling. Fresh the shoot and root samples were taken, and immediately frozen in liquid N_2_ and kept at -80 °C. A 25-mg sample was placed in a tube and extracted in 500 µL of acetonitrile: methanol: water (2:2:1) with an internal standard. Following a 30-sec vortex, the samples were homogenized at 35 Hz for 4 min, sonicated for 5 min in an ice-water bath, incubated for 1 h at 40 °C, then centrifuged for 15 min at 12,000 g at 4 °C. After transfer to a tube, 250 µL of supernatant was dried at 37 °C in a vacuum concentrator. After 10 min of sonication in an ice-water bath, the dried samples were combined with 200 µL of 50% acetonitrile, which was followed by 15-min centrifugation at 13,000 g under 4 °C. The supernatant (100 µL) was utilized for liquid chromatography–mass spectrometry analysis (LC-MS). Ultra-high-performance liquid chromatographic separation was performed using an Agilent 1290 Infinity series UHPLC System (Agilent Technologies, Santa Clara, CA, USA) equipped with a UPLC BEH Amide column (2.1 × 100 mm,1.7 μm, Waters). Six replicates for each treatment were determined [30]. Unsupervised principal component analysis was conducted using the statistics function, prcomp, within R. Differentially abundant metabolites between groups were determined by variable importance in projection (VIP) ≥ 1 and absolute log_2_(fold change) ≥ 1.

### Statistical analysis

Different letters indicate significant differences among means as determined using one-way ANOVA followed by Tukey’s HSD test (*P* < 0.05). Significant differences (*, *P* < 0.05; **, *P* < 0.01; ***, *P* < 0.001) were determined by Student’s *t*-tests between two groups using the SPSS 17.0.

## Results

### Physiological responses of tobacco plants to CNS

**Fig. 1:**
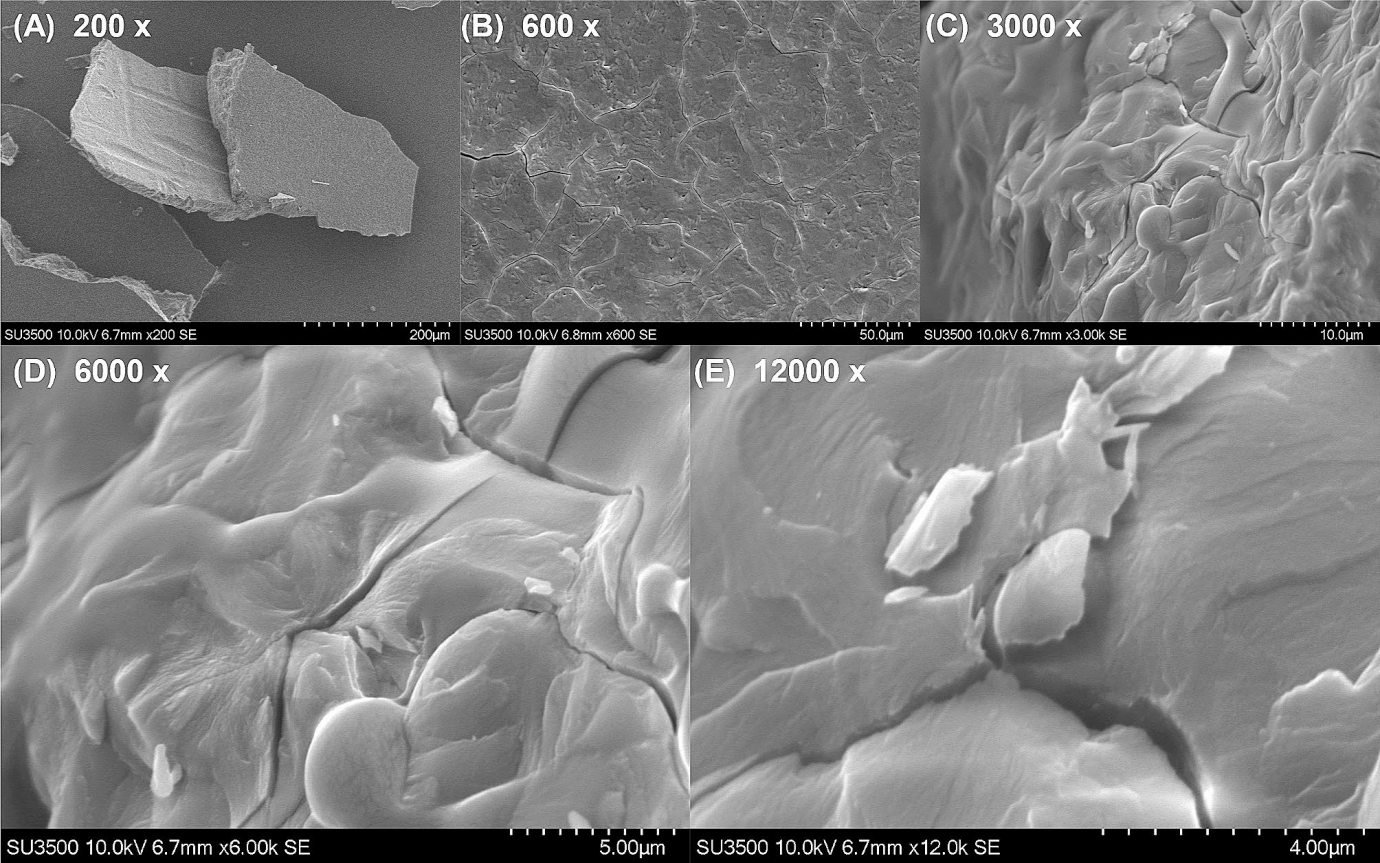
Scanning electron micrograph of carbon-nano sol (CNS). **(A-E)** 200- **(A)**, 600- **(B)**, 3,000- **(C)**, 6,000- **(D)**, and 12,000-fold **(E)** magnification of the CNS identified by scanning electron microscopy

**Fig. 2:**
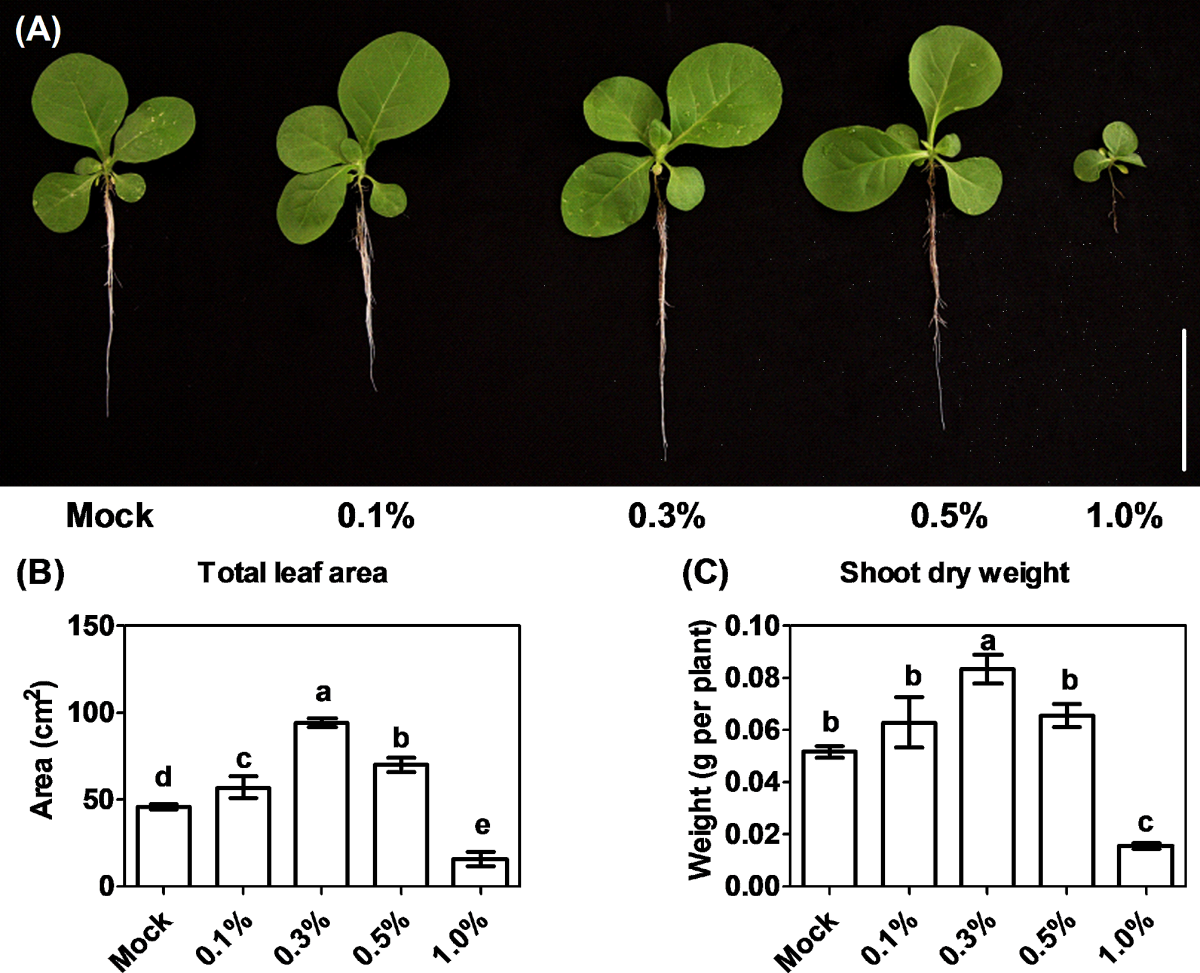
Effect of different concentrations of carbon-nano sol (CNS) on the growth of tobacco seedlings. **(A)** Growth performance of the tobacco seedlings at different CNS concentrations (scale bar = 7 cm); (B-C) Comparative analysis of leaf area **(B)** and shoot dry weight **(C)** of tobacco seedlings under different CNS concentrations. Mock: no CNS added; 0.1%, 0.3%, 0.5%, and 1.0% indicate the mass concentrations of exogenously added CNS. Data are presented as means (*n* = 5) ± SD. Different letters indicate significant differences among means as determined using one-way ANOVA followed by Tukey’s HSD test (*P* < 0.05)

Scanning electron microscopy showed that CNS presented foliated structure (Fig. 1A), which was magnified to show rugged appearance (Fig. 1B-E). To characterize the role of CNS in regulating the growth and development of tobacco plants, five CNS addition concentrations were implemented. Through systematic screening, the optimal concentration conducive to the promotion of tobacco plant growth was identified (Fig. 2). Comparative analysis revealed that, similar to the control (mock), 0.1% and 0.5% CNS additions did not significantly change the growth of tobacco seedlings. In contrast, the addition of 0.3% CNS exhibited a significant promotion in the growth of tobacco seedlings, while the addition of 1.0% CNS resulted in a significant inhibition of tobacco seedling growth (Fig. 2A). In addition, the 0.3% CNS also significantly increased the leaf areas (Fig. 2B) and shoot biomasses (Fig. 2C) of tobacco plants.

Through determination of the root physiological indexes and root system configuration, we found that 0.3% CNS addition significantly increased the root biomass of tobacco plants, 1.0% CNS addition significantly decreased the root biomass of tobacco plants, and the other two nanocarbon additions (0.1% and 0.5%) did not significantly change root biomass (Fig. S1 A). Additionally, CNS did not significantly alter the root/shoot ratio of tobacco plants (Fig. S1 B). Further determination of the root system configuration of tobacco seedlings revealed that the 0.3% CNS significantly increased the maximum root length (Fig. S1C), total root length (Fig. S1 D), root surface area (Fig. S1 E), root volume (Fig. S1 G), root tip number (Fig. S1 H), and average root diameter (Fig. S1 F) compared to CNS-free conditions (mock).

### Differential ionomic responses of tobacco plants to CNS

**Fig. 3:**
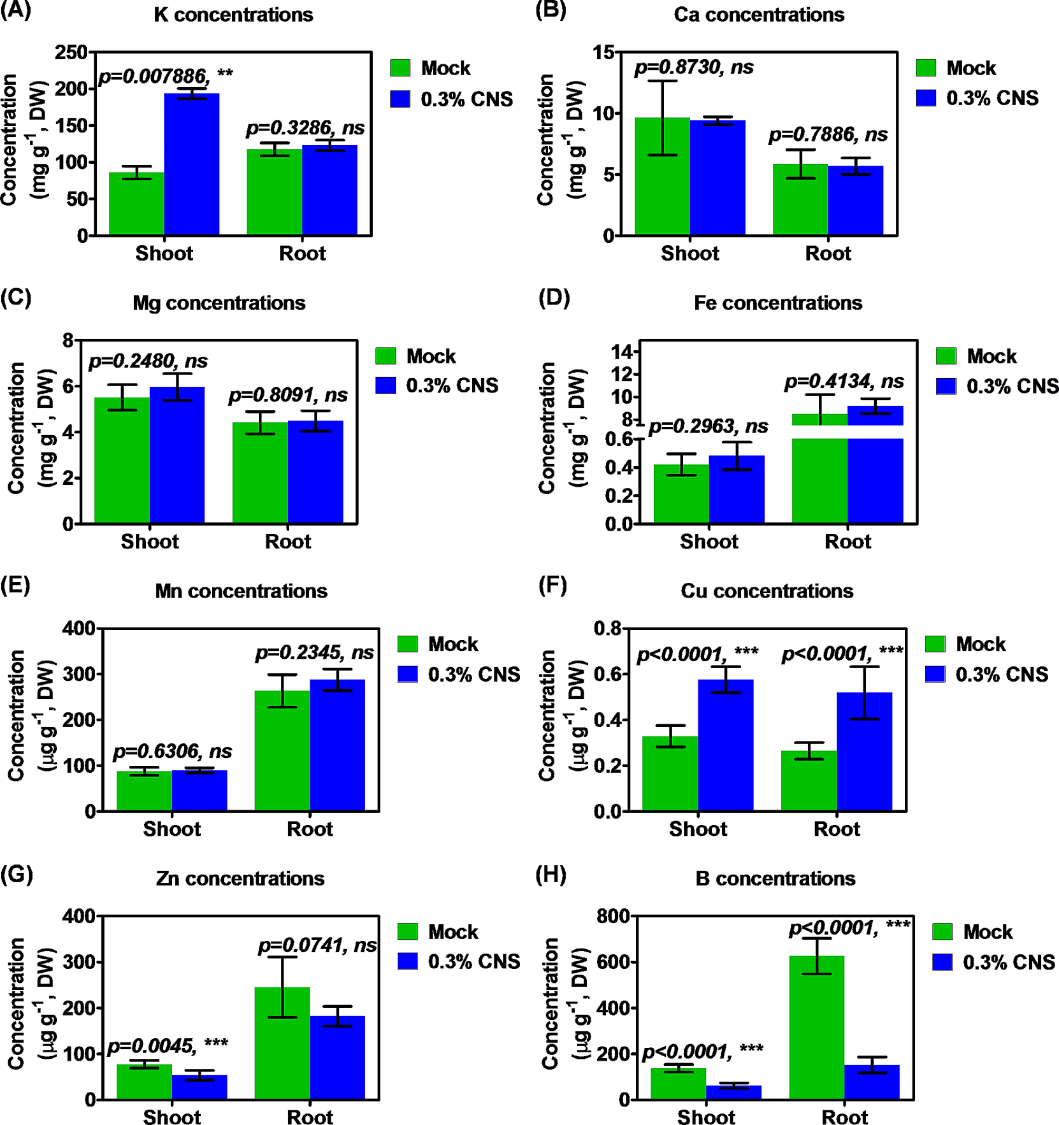
Comparative analysis of ion concentrations in tobacco seedlings grown under the control and 0.3% carbon-nano sol (CNS) conditions. (A-H) The concentrations of potassium/K **(A)**, calcium/Ca **(B)**, magnesium/Mg **(C)**, iron/Fe **(D)**, manganese/Mn **(E)**, copper/Cu **(F)**, zinc/Zn **(G)**, and boron/B **(H)**. Mock: no CNS added; 0.1%, 0.3%, 0.5%, and 1.0% indicate the mass concentrations of exogenously added CNS. Data are presented as means (*n* = 5) ± SD. Significant differences (*, *P* < 0.05; **, *P* < 0.01; ***, *P* < 0.001) were determined by Student’s *t*-tests between two groups using the SPSS 17.0

To elucidate the physiological mechanism underlying the growth promotion observed with 0.3% CNS, we examined the ionomic profiles of several mineral cations using ICP-MS. Compared with the control (mock), 0.3% CNS obviously increased shoot K concentrations, while it did not alter the K concentrations in the roots (Fig. 3A). However, 0.3% CNS did not significantly affect the concentrations of Ca (Fig. 3B), Mg (Fig. 3C), Fe (Fig. 3D), and Mn (Fig. 3E) in both shoots and roots of tobacco plants. There was only a notable increase in the Cu concentrations of both shoots and roots (Fig. 3F), while the concentrations of B and Zn were significantly reduced (Fig. 3G, H). Physiologically, the ionomic results suggested that 0.3% CNS may promote the growth of tobacco plants potentially through increasing root-to-shoot K translocation in tobacco plants.

### CNS induced rhizosphere acidification and influenced IAA homeostasis

**Fig. 4:**
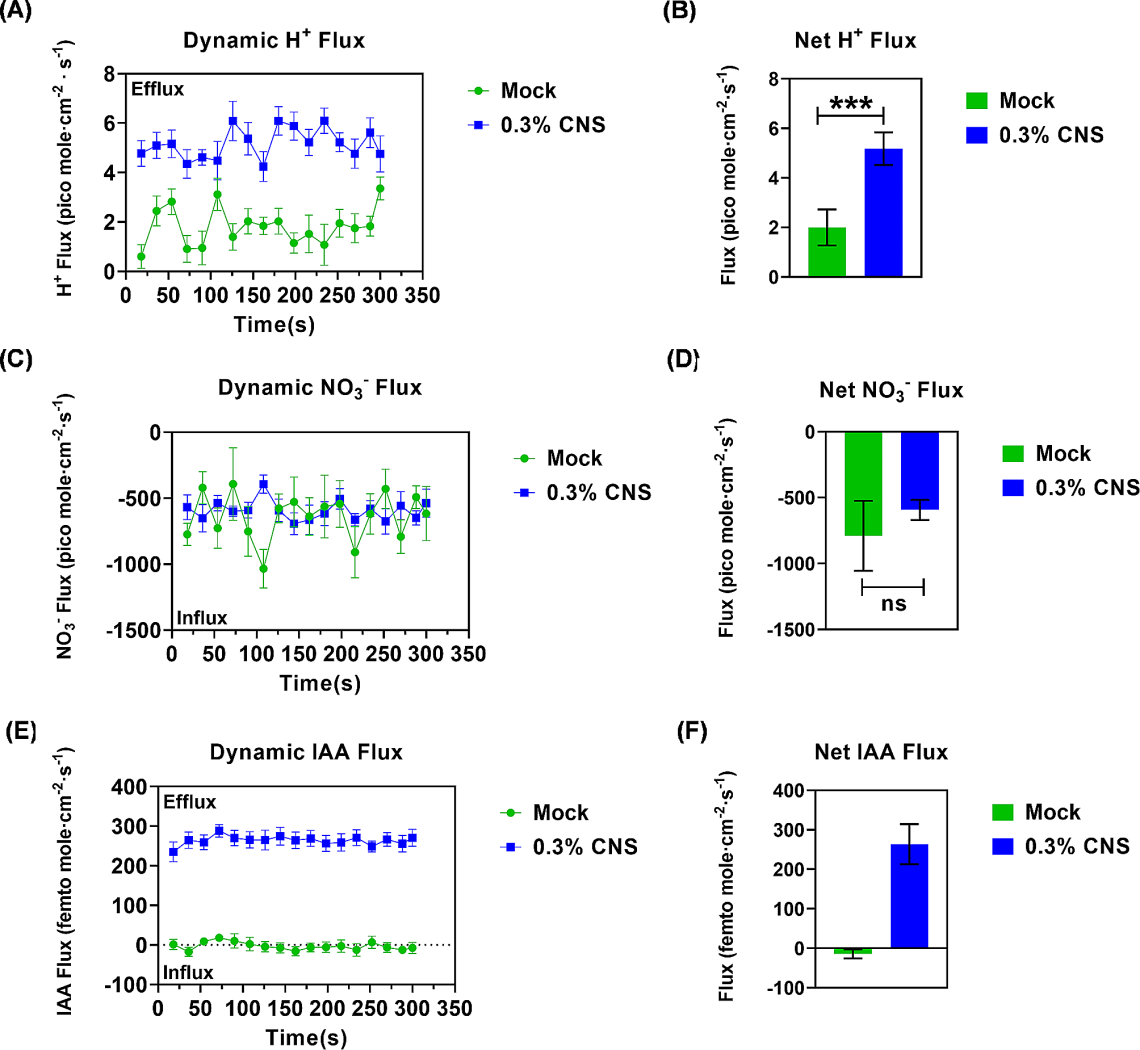
The net and dynamic flux rate of H^+^ and IAA in the primary roots of tobacco plants under the control and 0.3% carbon nano sol (CNS) conditions. **(A-F)** The net and dynamic flux of H^+^**(A, B)**, NO_3_^−^**(C, D)**, and IAA **(E, F)**. The positive and negative data indicate the efflux and influx rates of ions, respectively. The columns represent the mean efflux and net influx rate averaged over the entire 5 min (± SD, *n* = 8). Significant differences (*, *P* < 0.05; **, *P* < 0.01; ***, *P* < 0.001) were determined by Student’s *t*-tests between two groups using the SPSS 17.0

We further used the NMT technique to analyze the net flux dynamics in tobacco roots in response to 0.3% CNS conditions. Our NMT assays revealed that the net fluxes of H^+^, and NO_3_^−^, and IAA at the root surface varied under 0.3% CNS supply conditions along the root tips (Fig. 4). In response to the 0.3% CNS treatment, distinct alterations in ion flux dynamics were observed in tobacco roots. Specifically, under this treatment, the roots exhibited increased H^+^ efflux compared to conditions without CNS additions (Fig. 4A). Notably, there was no significant difference in the NO_3_^−^ influx between the two treatments (Fig. 4B). In addition, a noteworthy observation was the shift in the flux of IAA: under the CNS-free treatment, IAA exhibited influx, while under the 0.3% CNS condition, IAA flux transitioned to efflux (Fig. 4C).

### Differential metabolomic responses of tobacco plants to CNS

**Fig. 5:**
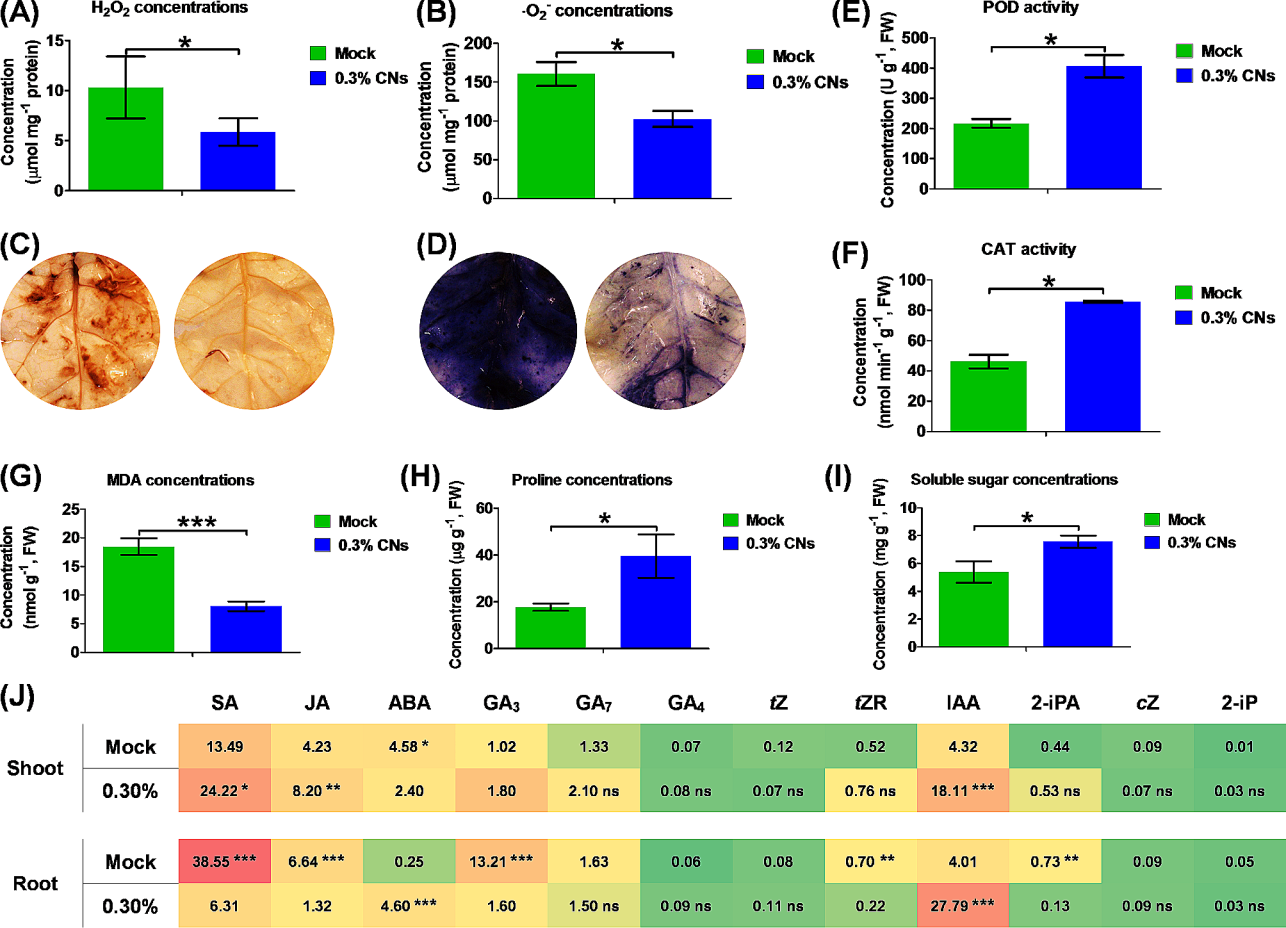
Phytohormone concentrations in the shoots and roots under the control and 0.3% carbon nano sol (CNS) treatment conditions. Data are presented as means (*n* = 5) ± SD. IAA, indole-3-acetic acid; *c*Z, *cis*-Zeatin; *t*Z, trans-Zeatin; iP, isopentenyladenine; *t*ZR, trans-zeatin riboside; GA, gibberellic acid; ABA, abscisic acid; JA, jasmonic acid; SA, salicylic acid. Significant differences (*, *P* < 0.05; **, *P* < 0.01; ***, *P* < 0.001) were determined by Student’s *t*-tests between two groups using the SPSS 17.0

We next examined the differences in ROS levels between the CNS-free and 0.3% CNS treatment, and found higher H_2_O_2_ and O_2_^−^ concentrations under the CNS-free condition (Fig. 5A, B), which was confirmed by the DAB and NBT staining (Fig. 5C, D). Analysis of the antioxidant enzyme activity showed that 0.3% CNS markedly increased the activity of both peroxidase (POD) and catalase (CAT) in the tobacco plants (Fig. 5E, F). Malondialdehyde (MDA) is the end product of the peroxidation of membrane lipids and can reflect the extent to which a plant suffers adversity. We found significantly higher concentrations of MDA under CNS-free than in 0.3% CNS condition (Fig. 5G). Proline, an important amino acid, is essential for maintaining plant metabolism and growth under abiotic stress conditions. We found significantly higher concentrations of proline under the CNS-free treatment than under the 0.3% CNS treatment (Fig. 5H), which also induced the accumulation of soluble sugar in tobacco plants (Fig. 5I).

Given that the greatly altered metabolomic profile afore-mentioned, we examined differentially accumulated metabolites (DAMs) in roots and shoots. The PCA analysis showed that exposure to CNS led to a distinct separation of metabolites in roots and shoots compared to CNS-free conditions (Fig. S2A), indicating a reprogramming effect induced by CNS. A total of 789 tobacco plant metabolites were identified, and 141 and 163 DAMs were identified in tobacco roots and shoots, respectively. Notably, 49 DAMs were found to be common to both roots and shoots. Furthermore, several of the top 20 DAMs were significantly enriched in the roots or shoots of tobacco plants (Fig. S2B-C).

To determine the effect of CNS on phytohormone homeostasis, we measured the concentration profiles of IAA combined with GA, ABA, JA, SA, *t*Z, *c*Z, *t*ZR, and 2-iPA in the roots and shoots (Fig. 5J). Significantly higher concentrations of SA, GA_3,_ and IAA were observed in the shoots under the 0.3% CNS treatment than under control, whereas no significant differences were detected for the other nine phytohormones. In the roots, both IAA, JA, GA_3_, and SA showed markedly higher concentrations under the 0.3% CNS treatment than under control, whereas the concentrations of SA and IAA under the 0.3% CNS were almost five folds higher than those under control. In addition, hormones such as *c*Z, GA_4_, *t*Z, *t*ZR, and 2-iPA were not detected in roots and shoots.

### Genome-wide transcriptional responses of tobacco plants to CNS

**Fig. 6:**
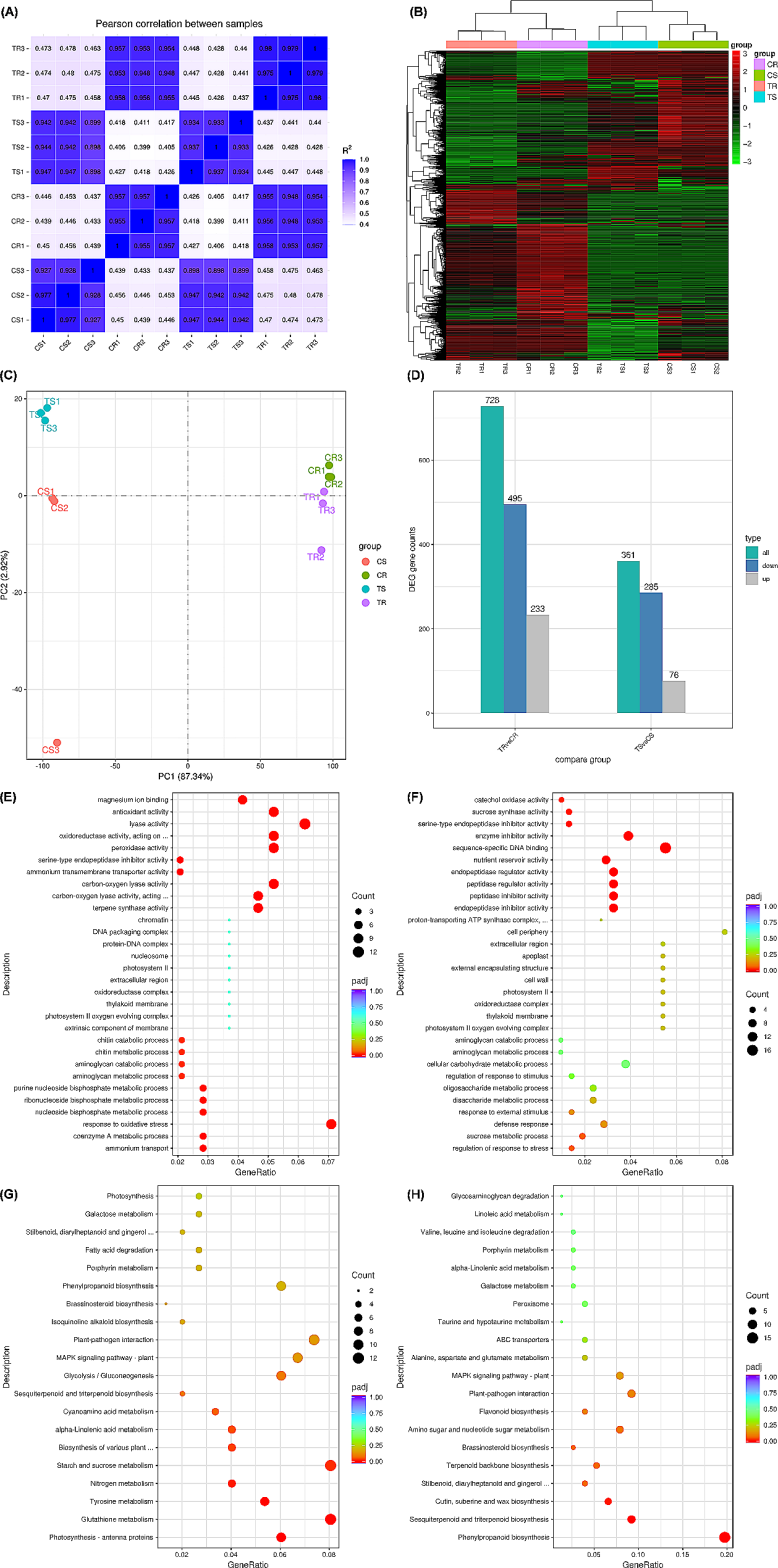
Clustering analysis of differentially expressed genes (DEGs) in the shoots and roots of tobacco plants under the control and 0.3% carbon nano sol (CNS) treatments. **(A)** Pearson correlation coefficients of the RNA-seq data between each pair of biological replicates. S and R indicate shoots and roots; **(B)** Clustering trees among the three biological replicates of each sample; **(C)** Principal component analysis of genome-wide differential gene expression profiling in the shoots and roots of tobacco plants under different carbon-nano sol addition concentrations. **(D)** Statistics of the number of differentially expressed genes. C: Mock; T: 0.3% CNS. S: Shoot; R: Root. Gene ontology (GO) enrichment and KEGG pathway enrichment analysis of different expressed genes (DEGs) in tobacco plants under varying NPs abundances. **(A-B)** Highly accumulated GO terms in the shoots **(A)** and roots. **(C-D)** Highly accumulated KEGG pathways in the shoots **(C)** and roots **(D)**

To investigate the molecular responses of tobacco to varying abundances of CNS, we conducted a high-throughput genome-wide transcriptome sequencing for both shoots and roots of tobacco seedlings subjected to control (mock) and treatment (0.3% CNS) conditions. Each set of samples consisted of three biological replicates; yielding a total of 936,176,322 raw reads (∼ 140.43 Gb) obtained across the 12 samples. After removing adapter sequences and low-quality reads, a total of 75,412,509 clean reads (∼ 11.31 Gb) were obtained from each sample. The quality assessments revealed a Q_20_ > 97% and Q_30_ > 93% for the clean reads from all 24 samples, and the GC content across all samples was about 42.95%. The majority of the *Pearson* correlation coefficients between each pair of biological replicates were higher than 0.90 (Fig. 6A). Clustering trees depicted comparable heights among the three biological replicates of each sample. Hierarchical clustering of genome-wide gene expression exhibited similar expression patterns among the three biological replicates of each sample (Fig. 6B). Principal component analysis (PCA) underscored significant distinctions in transcriptomic features between shoots and roots under the two treatment conditions (Fig. 6C). In the roots, a total of 728 genes exhibited differential expression under the 0.3% CNS condition compared to the CNS-free (mock) condition. Similarly, in the shoots, 361 genes demonstrated differential expression under the 0.3% CNS condition relative to the control (Fig. 6D).

The gene ontology (GO) enrichment analysis of functional significance allowed us to distinguish major biological functions of the DEGs under varying CNS abundances (Fig. 6E, F). The highly enriched GO terms revealed significant involvement in oxidative stress response, antioxidant activity, and oxidoreductase activity in the shoots. In contrast, the CNS-responsive DEGs in the roots were primarily associated with sequence-specific DNA binding categories. To further identify the biological pathways active in tobacco during exposure to CNS abundances, we characterized the pathways using the KEGG database (Fig. 6G, H). The KEGG analysis showed that glutathione metabolism, starch and sucrose metabolism, and photosynthesis were enriched under the 0.3% CNS treatment.

### Genome-wide identification of *NtNPF* family genes

Previous studies have suggested that nitrate peptide family (NPF) transporters have multiple transport activity of various substrates, such as nutrients and phytohormones, and are widely involved in the biological processes [29]. Therefore, to reveal the core genes that are involved in the CNS-mediated tobacco plant growth, we focused on the *NPF* family genes in tobacco. We utilized sequences of 143 NtNPF proteins and 53 AtNPF proteins to construct a phylogenetic tree to elucidate the evolutionary links and functional divergence among NPF proteins in tobacco (Fig. S3, Table S2). Overall, the number of *NPF* homologs in *N. tabacum* was more than twice that found in *A. thaliana*.

As in the previous study, the candidate NPFs were split into eight subfamilies based on the topologies and bootstrap support values of the NJ phylogenetic tree [31]. The distribution of *NtNPFs* among the subfamilies was as follows: *NtNPF1s* (20 members), *NtNPF2s* (25 members), *NtNPF3s* (4 members), *NtNPF5s* (25 members), *NtNPF6s* (20 members), *NtNPF7s* (11 members), and *NtNPF8s* (14 members). There was a clear tendency of expansion among the eight subfamilies, as seen by the variations in the number of *NtNPFs* within them (Fig. S3). To further clarify the potential functions of *NPFs* in *N. tabacum*, we employed the MEME tool to identify 10 conserved motifs (Fig. S4A). The motif analysis showed that most *NtNPFs* contain motif1-motif 10. However, certain genes exhibit variations in motif presence. Notably, NtNPF7.3 lacks motif 10, while NPF6.14 and 6.18 only have motif 1 and motif 10, and NtNPF7.9 is devoid of motif 3, motif 6, and motif 9. In addition, motif patterns within a subgroup of *NtNPFs* are similar. To identify the domains of the *NPF* family proteins in *N. tabacum*, the protein sequences of 143 genes were submitted to NCBI for domain prediction. The results, visualized using the TBtools software, revealed a total of 143 genes contained two conserved domains–the major facilitator superfamily (MFS) and peptide transport 2 superfamily (PTR2) (Fig. S4B).

To evaluate the exon-intron organization diversity of *NtNPFs*, the gene structures of each *NtNPF* were delineated (Fig. S5). Most of the *NtNPFs* had four exons and three introns, and several genes had five exons and four introns, while *NtNPF1.9* contained 12 exons and *NtNPF1.2* contained one exon. Similarly, the majority of *NtNPFs* in the same subgroups generally had similar gene structures. In addition, the intron lengths are slightly different among different *NtNPFs*. Relative to *NtNPF7.4*, the introns within *NtNPF7.8* were large. Although the exon-intron structures of most closely related genes exhibited high similarity and conservation, there were several differences.

The unequal distribution of genes on the chromosomes may facilitate the exchange of sequences via recombination or mispairing. We analyzed the distribution of the identified 143 members in *NPF* families on the 24 chromosomes of *N. tabacum* and found it was not uniform (Fig. S6A). Evidently, *NtNPF5.2* is only on the chromosome Nt03, and *NtNPF4.18* on Nt17, while the chromosomes of Nt04, Nt09, Nt18, and Nt20 have the most genes. *NtNPFs* on different chromosomes are located far away from one another, and the distribution of genes on chromosomes is rather dispersed. To better understand the evolution of *NtNPF*s, the synteny of the *NPF* pairs between the genomes of *N. tabacum* and *A. thaliana* was constructed (Fig. S6B). We found that 27 *NtNPF*s exhibited syntenic relationships with *AtNPF*s. Some At*NPF*s were associated with more than one orthologous copy in *N. tabacum*. For example, *AtNPF1.1* (*AT3G16180*) showed a syntenic relationship with *NtNPF1.13*, *NtNPF1.14*, and *NtNPF1.16* (Table S3).

The plantCARE database was used to search for CREs in the 2,000 bp upstream promoter region sequences of each *NPF* gene in order to examine the potential regulatory mechanisms underpinning *NPFs* in response to abiotic stressors and hormones. We found that the promoter regions of each *NtNPF* contain several stress- and hormone-related CREs. A total of 28 CREs were detected, including 945 light-responsive CREs, 204 MeJA-responsiveness CREs, 188 abscisic acid responsiveness CREs, 115 anaerobic induction CREs, and 82 GA-responsive CREs (Fig. S7). The most *cis*-acting elements found in the promoter regions of the 143 *NPF* genes were light-responsive CREs (945) and the fewest were in the part of gapA (gapA-CMA1) involved with light responsiveness CREs (2) (Table S3).

### Molecular characterization the core gene(s) involved in the CNS-promoting growth of tobacco plants

**Fig. 7:**
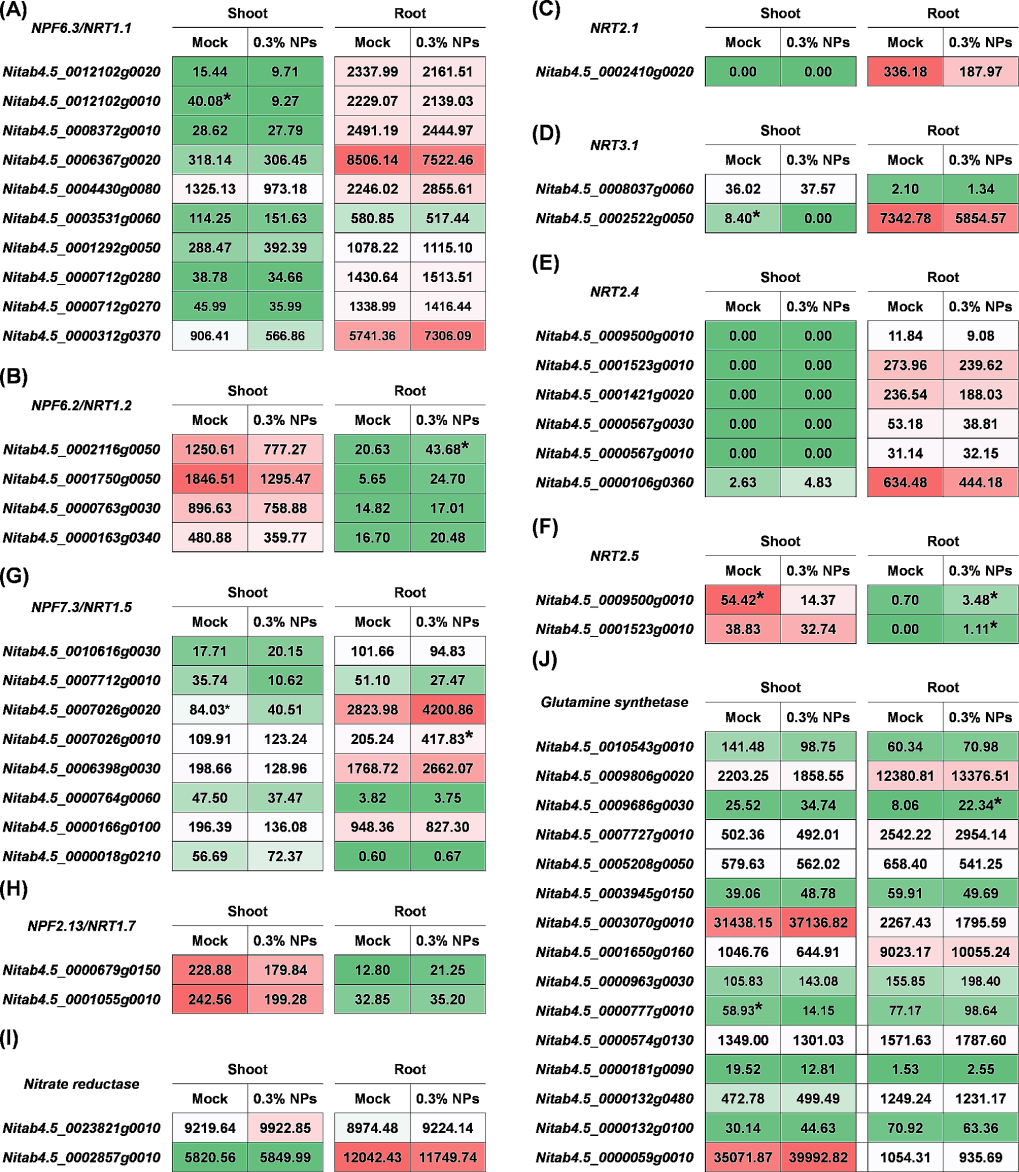
Transcriptional profiling of the genes associated with nitrogen (N) transport and metabolism under the control and 0.3% carbon nano sol (CNS) treatments. Expression profiling of *NPF6.3/NRT1.2***(A)**, *NPF6.2/NRT1.2***(B)**, *NRT2.1***(C)**, *NRT3.1***(D)**, *NRT2.4***(E)**, *NRT2.5***(F)**, *NPF7.3/NRT1.5***(G)**, *NPF2.13/NRT1.7***(H)**, *nitrate reductase/NR***(I)**, and *glutamine synthetase/GS***(J)** in the roots and shoots. The heatmap shows gene expression levels as indicated by FPKM values. The differentially expressed genes with higher expression between the CNS-free condition and 0.3% CNS are denoted by asterisks

In this study, we investigated the expression of genes related to efficient nitrogen (N) uptake, transport, and N assimilation. *NRT1.1/NPF6.3*, postulated to be a “transceptor” (transporter/receptor) with dual NO_3_^−^ transport and sensing functions [32], did not exhibit significant alterations in the transcript levels under the 0.3% CNS treatment (Fig. 7A). In addition, the expression of the genes related to other nitrate transporter proteins responsible for nitrate uptake, including *NPF6.2/NRT1.2*, *NRT2.1*, *NRT3.1*, *NRT2.4*, *NRT2.5*, remained unaffected by the 0.3% CNS treatment (Fig. 7B-F**).** The transcript levels of *NPF2.13/NRT1.7*, which is responsible for the source-to-sink remobilization of nitrate, showed no significant changes (Fig. 7H). However, the *NPF7.3/NRT1.5* transporter, responsible for xylem loading of nitrate/K^+^ in the roots and subsequent transport to the shoot, displayed increased nitrate content in root xylem wounding fluid under the 0.3% CNS treatment. This observation correlated with a significant up-regulation in the expression of *NtNPF7.3/NtNRT1.5* (*Nitab4.5_0007026g0010*) (Fig. 7G). Moreover, the expression of *NtNPF2.13/NtNRT1.7*, responsible for nitrate remobilization from sources to sink organs, was also not significantly changed under the 0.3% CNS treatment (Fig. 7H). Nitrate reductase (NR) and glutamine synthetase (GS) are the major enzymes in the process of N assimilation. Notably, the transcript levels of NR and GS remain unaltered under the 0.3% CNS treatment (Fig. 7I, J). A previous study has reported that Arabidopsis NPF7.3/NRT1.5 protein functions as a transporter of indole-3-butyric acid (IBA), a precursor of the major endogenous IAA [33].Building on these findings, we hypothesized that *NtNPF7.3/NtNRT1.5* (*Nitab4.5_0007026g0010*)-mediated K^+^ and IAA translocation may be the key physiological and molecular mechanism by which 0.3% CNS promote the growth of tobacco plants.

### Transcriptional responses of genes involved in ROS production, scavenging, and phytohormone biosynthesis to CNS

NADPH oxidase/respiratory burst oxidase homolog (RBOH) proteins produce localized bursts of reactive oxygen species (ROS) that modulate growth, development, and stress responses in plants [34]. Most *RBOH* DEGs were upregulated in the shoots or roots under 0.3% CNS (Fig. S8A). Among the antioxidant enzyme genes, *NtAPX* showed higher expression levels and fold changes under 0.3% CNS, shedding light on its potential role as a contributor to ROS scavenging under 0.3% CNS (Fig. S8B). The concentrations of IAA, SA, JA, and GA were markedly increased under the 0.3% CNS treatment (Fig. S8). Therefore, we investigated transcriptional responses of the phytohormone metabolism-related genes to CNS in tobacco. In auxin synthesis, *YUCCA* and *TAA/TAR* genes play pivotal roles. Notably, under the 0.3% CNS treatment, the expression levels of most *NtYUCCAs* were significantly up-regulated in the roots. Particularly, *NtYUCCA6* (*Nitab4.5_0003956g0030*) displayed enhanced expression levels and fold changes, potentially suggesting a key role in IAA biosynthesis (Fig. S8C). Two SA biosynthesis pathways have been elucidated in plants, namely, the ICS and the PAL pathways [35]. Under the 0.3% CNS treatment, only the *NtPAL* (*Nitab4.5_0018680g0010*) gene was down-regulated in the shoots and roots (Fig. S8D). GA biosynthesis starts with geranylgeranyl diphosphate, which is then transformed into GA by *CPS*, *KS*, *EKO*, and *GA 7/13/20/3/2-oxidases* [36]. Under the 0.3% CNS treatment, only the expression levels of *NtGA*_*20*_*-oxidases* were significantly different in the shoots and roots (Fig. S8E). The JA biosynthesis pathway involves the oxygenation of fatty acids generated from lipids by a series of enzyme genes, including *PLA1*, *LOX*, *AOS*, *AOC*, *OPR*, and *JAR1* [35]. There were no significant differences in *AOC*, *OPR*, and *JAR1* under the 0.3% CNS treatment. The majority of DEGs related to *NtPLA, NtAOS*, and *NtLOX* exhibited significant down-regulation in the tobacco plants (Fig. S8F). Among the numerous DEGs, the genes involved in nutritional ion homeostasis were given special attention since they were critical for tobacco plants under the 0.3% CNS treatment. In general, most phosphate (Pi) transporter genes did not exhibit a consistent response, *NtPHT1;1* (*Nitab4.5_0000436g0220*) showed decreased expression levels, while *NtPHT1;4* (*Nitab4.5_0009483g0010*) and *NtPHT2;1* (*Nitab4.5_0000370g0100*) showed enhanced expression levels (Fig. S8G). The majority of the transporter genes (including *COPTs* and *MGTs*) involved in the uptake and transport of Cu^2+^ and Mg^2+^ showed obvious upregulation in the roots under the 0.3% CNS treatment (Fig. S8H, I). The transcriptomic results showed that most of the K^+^ transporter genes, including high-affinity K^+^ transporter type (*HKT*) responsible for root xylem Na^+^ unloading and the plasma membrane-localized K^+^ influx transporter genes (*AKT/KAT*) were downregulated under the 0.3% CNS treatment (Fig. S8J).

### A proposed model for CNS-promoting the growth of tobacco plants

**Fig. 8:**
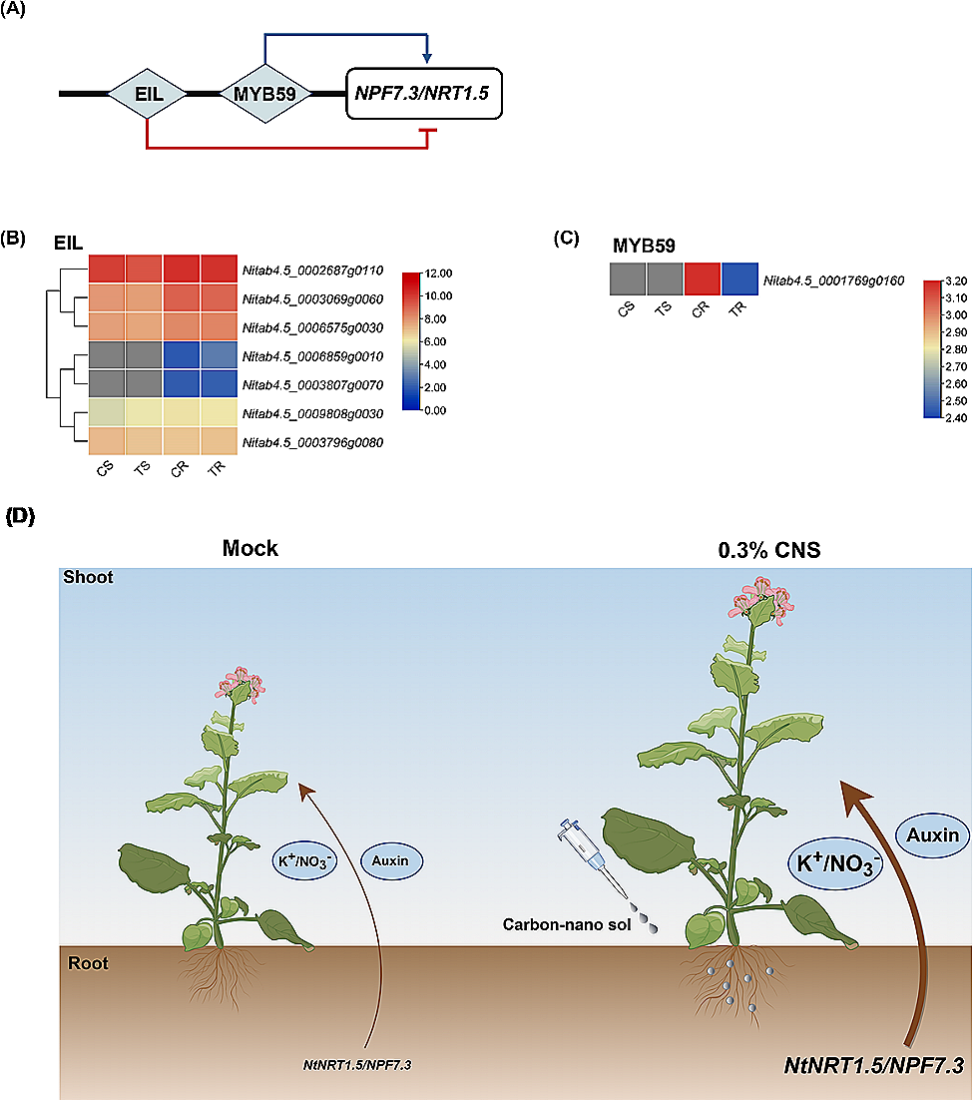
Transcriptional profiling of potential transcription factors regulating the expression of *NtNPF7.3/NtNRT1.5* and a proposed model for the mechanism underlying carbon nano sol (CNS)-promoting the growth of tobacco plants. **(A)** Reported EIN3/EIL-mediated negative and MYB59-mediated positive transcriptional regulation of the *NPF7.3/NRT1.5* expression in *Arabidopsis thaliana*. **(B-C)** Transcriptional profiling of the transcription factor genes *EIN3/EIL* and *MYB59* in the shoots (S) and roots (R) under CNS-free **(C)** and 0.3% CNS treatment (T). **(D)** A proposed model for the mechanism underlying CNS-promoting the growth of tobacco plants. CNS might motivate an unknown sensor, which further affected the protein activity or abundances of EIL/EIN and MYB59. Then, EIL/EIN or MYB59 regulated the transcriptional expression level of *NtNPF7.3/NtNRT1.5*, which might promote the translocation of K^+^, NO_3_^−^, and IAA from roots to shoots, facilitating photosynthesis and plant growth. The larger the font sizes, the higher the K^+^/NO_3_^−^/IAA content and the *NtNPF7.3/NtNRT1.5* expression

Building on previous studies proposing ethylene and JA signaling mediate the downregulation of *NRT1.5* via *EIN3/EIL1*, and *MYB59* directing root-to-shoot K^+^/NO_3_^−^ transport by positively regulating the expression of *NPF7.3/NRT1.5* in Arabidopsis (Fig. 8A), we sought to explore whether *NPF7.3/NRT1.5* upregulation under CNS supply was similarly orchestrated by *EIN3/EIL1* and *MYB59* in tobacco plants. We found significantly higher expression levels of *NtEIN3s/NtEILs* under the 0.3% CNS treatment (Fig. 8B). Interestingly, *NtMYB59* displayed an expression preference opposite to *NtNPF7.3/NtNRT1.5*, undergoing down-regulation under the 0.3% CNS treatment (Fig. 8C). In light of these findings, we speculated that *NtMYB59* and *NtEIL* regulate the expression of *NtNPF7.3/NtNRT1.5*, while the regulation is contrary to that reported in Arabidopsis. Therefore, we proposed that CNS might motivate an unknown sensor, which further affected the protein activity or abundances of NtEIL/EIN and NtMYB59. Then, EIL/EIN or NtMYB59 regulated the transcriptional expression level of *NtNPF7.3/NtNRT1.5*, which might promote the translocation of K^+^, NO_3_^−^, and IAA from roots to shoots, facilitating photosynthesis and plant growth (Fig. 8D).

## Discussion

Previous studies have highlighted the efficacy of nanomaterials in enhancing nutrient uptake, resisting abiotic stress, and photosynthesis in tobacco plants [37–41]. Specifically, carbon nanotubes have been shown to boost tobacco cell growth by increasing the expression of the aquaporin gene (*NtPIP1*) [9]. In tobacco BY-2 cells, carbon nanoparticles have demonstrated the ability to increase the expression of K^+^ transporter genes, thereby enhancing K^+^ accumulation [18]. CNS has also been associated with heightened K^+^ uptake in the roots and increased K^+^ accumulation by influencing the expression of K^+^ channel genes in the root system of tobacco plants [37]. Addressing the challenges posed by Cd toxicity, which severely suppresses tobacco development and ion balance, foliar spraying with Fe_3_O_4_ and ZnO CNS has been found to alleviate the adverse effects of Cd toxicity on tobacco growth [16]. Water-soluble daphnetin nanoparticles, specifically those grafted with carboxymethyl chitosan, have demonstrated effective suppression of tobacco bacterial wilt in pot experiments [38]. In addition, Cu composite rod-like nanoparticles have exhibited the ability to protect tobacco by inducing disease resistance [39]. Chiral Cu sulfide nanoparticles with a size of 3 nm have shown site-selective cleavage of capsids in tobacco mosaic virus under sunlight, thereby inhibiting viral infectivity [40]. Tobacco, recognized as a crucial model plant with diverse applications in transgenic techniques, benefits from nanomaterials in various ways. Carbon nanocarriers, for instance, can efficiently deliver small interfering RNA to intact tobacco plant cells, facilitating effective gene knockdown [41]. In summary, nanomaterials play a pivotal role in promoting tobacco plant growth through diverse mechanisms.

Numerous studies have underscored the impact of nanomaterials on agricultural ecosystems, often characterized as a “low-promoting and high-suppressing” dynamic, thereby contributing to the overall health of the agricultural environment [13]. In this study, CNS exhibited a dual effect on the growth performance of tobacco plants: enhancing it at moderate concentrations while inhibiting it at much higher concentrations (Fig. 2). Notably, water-soluble carbon nano-dots have been previously reported to promote root development of wheat plants [42]. In our study, the 0.3% CNS treatment facilitated the growth of both shoots and roots (Fig. 2, S1). Considering the crucial role of nutrient accumulation, distribution, and balance in maintaining plant growth, we tested ion concentration alterations in response to the additional 0.3% CNS treatment. The general profiles of the cations were not markedly changed between the mock and the 0.3% CNS treatment, which only induced the increase in the K and Cu concentrations but led to the decrease in the Zn and B concentrations (Fig. 3). Therefore, we proposed that the growth-promoting role of CNS might attributed to the concentration-increased nutrients (particularly K) not the reduced ones. Many studies have emphasized the interactive complexity of ions, particularly the ions with same charges and similar physio-chemical characteristics (such as Fe, Mn, Cu, and Zn). The hallmark of this relationship in plants is the increased accumulation of Cu in plant tissues under Fe deficiency and the overaccumulation of Fe under Cu deficiency. In summary, the differential profiles of Fe/Mn/Cu/Zn concentrations might be related to their complex interactions under additional CNS.

In Arabidopsis, endogenous auxin controls apoplastic acidification and the onset of cellular elongation in roots [43]. We observed that the efflux and concentration of IAA were increased in the roots, and the root H^+^ efflux increased accordingly under the 0.3% CNS conditions (Fig. 4). These results suggested a potential mechanism wherein IAA induces an upregulation of H^+^ efflux, thereby promoting the emergence or elongation of lateral roots in tobacco plants grown under the 0.3% CNS treatment. Plant hormones, particularly indole-3-acetic acid, play pivotal roles in regulating root development, and our findings support the hypothesis that IAA plays a key role in the promotion of tobacco growth under the 0.3% CNS conditions. In addition to IAA, we also found that 0.3% CNS increased GA and SA concentrations in both shoots and roots, and increased JA concentration in the roots. GA can regulate plant height and is famous for “Green revolution”. SA and JA are widely recognized as plant defense hormones. A previous study showed that the nano-selenium treatment enhances sugarcane resistance to *Xanthomonas albilineans* infection through increasing JA accumulation and the expression of JA biosynthesis-related genes [44]. Whether 0.3% CNS can regulate tobacco height and defense for biotic stresses through affecting these three phytohormones will be explored in the near future.

Plants employ intricate strategies, including molecular regulation and signal transduction, to promote their growth. N is an essential macronutrient for plant growth and development, with plant nitrate transporters categorized into the *NPF* (former ‘low-affinity’ NRT1/PTR) and ‘high-affinity’ *NRT2* families [45]. Previous studies have identified the ubiquity of the NPF family across plant species, such as 53 *NPFs* in Arabidopsis, 109 in the tea plant, and 40 in rice [46, 47]. In this investigation, a total of 143 *NtNPFs* were identified (Table S1). Existing research underscores the significance of *NtNRT1.1/1.2/2.1* in nitrate^−^ uptake and sensing in root systems [48]. Under the 0.3% CNS treatment, *NtNRT1.1* and *NtNRT2.1* exhibited preferential transcription in the roots. However, except for significant up-regulation of *NtNPF6.2* (*Nitab4.5_0002116g0050*), there were no notable differences in *NtNRT1.1/1.2/2.1* genes. Inorganic N assimilation is driven by N-acquiring enzymes such as nitrate reductase (NR), nitrite reductase (NiR) and glutamine synthetase (GS) [49] The present study showed altered expression only in *NtGlnA1* (*Nitab4.5_0009686g0030*) and *NtGlnA* (*Nitab4.5_0000777g0010*) under the 0.3% CNS treatment. *NRT1.5*, responsible for nitrate allocation to roots and stress tolerance [50], also functions as a transporter of indole-3-butyric acid (IBA), a precursor of the major endogenous IAA. IAA, a vital plant hormone synthesized from tryptophan via TAA/TAR and YUCCA proteins [51], exhibited increased expression in *NtNRT1.5* (*Nitab4.5_0007026g0010*) in the roots under the 0.3% CNS. In summary, our findings suggest that the increased expression of *NtNRT1.5* and *NtYUCCA6* (*Nitab4.5_0003956g0030*) may underlie the promotion of tobacco plant growth by the 0.3% CNS. Based on the differential expression profile of *NtEIN3s/EILs* and *NtMYB59* between the CNS-free and the 0.3% CNS treatment (Fig. 8), we proposed a potential involvement of *NtEIN3s/EILs* and *NtMYB59* in the transcriptional regulation of *NtNPF7.3/NRT1.5* based on two previous related studies [52, 53]. However, the model proposed by this study suggests that MYB59 negatively regulates *NtNPF7.3/NtNRT1.5* expression in tobacco plants, contradicting its positive regulatory role reported in Arabidopsis. Therefore, this discrepancy remains to be addressed with further investigation and exploration of potential species-specific differences or alternative regulatory mechanisms in the next work.

Under biotic and abiotic stresses, plants maintain homeostasis mainly by reallocating and adjusting a series of metabolic networks. In the present study, CNS induced higher metabolites in the shoots than in the roots. Lipids, as important compounds in living organisms, play a crucial role in regulating plant responses to various abiotic and biotic stresses [54]. CNS indeed markedly increased Cer (t18:0/16:0) and LacCer (d18:1/22:0) (belongs to sphingolipid) levels in the roots or shoots. We also found that 3-methyluridine (modified nucleosides of RNA) was induced by CNS in the shoots (Figure S2). mRNA modification regulation is a critical and pervasive biological mechanism driving major plant developmental processes such as embryo development, shoot stem cell destiny, and root development [55]. These results indicated that CNS induced metabolome profiling conferred a distinct mechanistic response to CNS in the roots and shoots, which was closely related to the occurrence of the direct interaction with CNS between tobacco organs.

Many studies on the effect of nanomaterials, including the CNS used in this study, on the growth and development of plants have been conducted in soil-less media, such as vermiculite, hydroponic culture, and agar medium. Under these circumstances, the supplemental nanomaterials are highly available with enhanced nanomaterials dissolution kinetics compared to soil for both host plants and rhizobia. Given that hydroponic culture systems provide limited information about the CNS-mediated promoting plant growth occurring in the real environment/agrosystems, to safeguard these ecologically significant relationships, large-scale application of CNS in farming systems should be preceded by long-term field trials in the next work. Nanomaterials with their unique physicochemical properties find extensive applications in agriculture production and other fields. Agricultural soils are exposed to a potential sink for ever-increasing amounts of different nanomaterials that inevitably enter the environment. However, concerns about their potential overuse and bioaccumulation put forward uncertain questions about their influence on animal health and environment safety. Biological roles and physicochemical interactions create pathways through which nanoparticles result in high biotoxicity. The integration of nanotechnology and environmental sustainability principles leads to the production of ecofriendly nanoparticle synthesis. Employing robust risk assessment methodologies, including the risk allocation framework, is recommended to address potential dangers associated with nanotechnology utilization.

## Conclusions

In this study, CNS was employed to establish an optimal system aimed at promoting the growth of tobacco plants. The study delved into comprehending the absorption and transport processes of this sol in tobacco plants, seeking to uncover the mechanisms that govern tobacco quality and resistance. The insights gained from this investigation provide evidence supporting the CNS use as a viable strategy for enhancing crop growth and improving overall crop quality. Moreover, the effect of CNS on the growth of tobacco plants at the whole growth stages remain to be further explored in the future, which will provide stronger evidence for the use of CNS to improve crop growth.

### Electronic supplementary material

Below is the link to the electronic supplementary material.

Supplementary Material 1

## Data Availability

All the data supporting the findings of this study are available within the paper and within its supplementary data published online. The raw data of the high-throughput transcriptome sequencing have been deposited in the NCBI Sequence Read Archive (SRA) under the Bioproject accession no. PRJNA995614.

## Abbreviations

CNS: Carbon nano sol
NMT: Non-invasive micro-test
NUE: Nitrogen use efficiency
MDA: Malondialdehyde
TBA: Thiobarbituric acid
H_2_O_2_: Hydrogen peroxide
IAA: Indole-3-acetic acid
IBA: Indole-3-butyric acid
*t*Z: Trans-zeatin
GA_3_: Gibberellin
ABA: Abscisic acid
JA: Jasmonic acid
SA: Salicylic acid
TPM: Transcripts per million reads
DEG: Differentially expressed gene
BLAST: Basic local alignment search tool
NJ: Neighbor-joining
MEGA: Molecular evolutionary genetics analysis
ROS: Reactive oxygen species
POD: Peroxidase
CAT: Catalase
DAMs: Differentially abudant metabolites
NPF: Nitrate peptide family
MFS: Major facilitator superfamily
PTR: Peptide transport superfamily
CREs: *cis*-regulatory elements
N: Nitrogen
NR: Nitrate reductase
NiR: Nitrite reductase
GS: Glutamine synthetase
