# Supplementary material for: Integrated analyses of ionomics, phytohormone profiles, transcriptomics, and metabolomics reveal a pivotal role of carbon-nano sol in promoting the growth of tobacco plants
Source: BMC Plant Biol. 2024 May 30;24:473. doi: 10.1186/s12870-024-05195-1 (PMC11137978; doi:10.1186/s12870-024-05195-1)
Supplement: Supplementary file 1 — Supplementary Material 1 [file 12870_2024_5195_MOESM1_ESM.docx]

**Supplementary files**

**Figure S1** The root system architecture of tobacco seedlings hydroponically cultivated under different carbon-nano sol (CNS) concentrations. (A-H) Root dry weight (A), root/shoot ratio (B), maximum root length (C), total root length (D), root surface area (E), average root diameter (F), root volume (G), and root tip number (H) in tobacco seedlings at different CNS concentrations. Mock: no CNS added; 0.1%, 0.3%, 0.5%, and 1.0% indicate the mass concentrations of exogenously added CNS, respectively. Data are presented as means (n=5) ± SD. Different letters indicate significant differences among means as determined using one-way ANOVA followed by Tukey’s HSD test (*P* < 0.05).


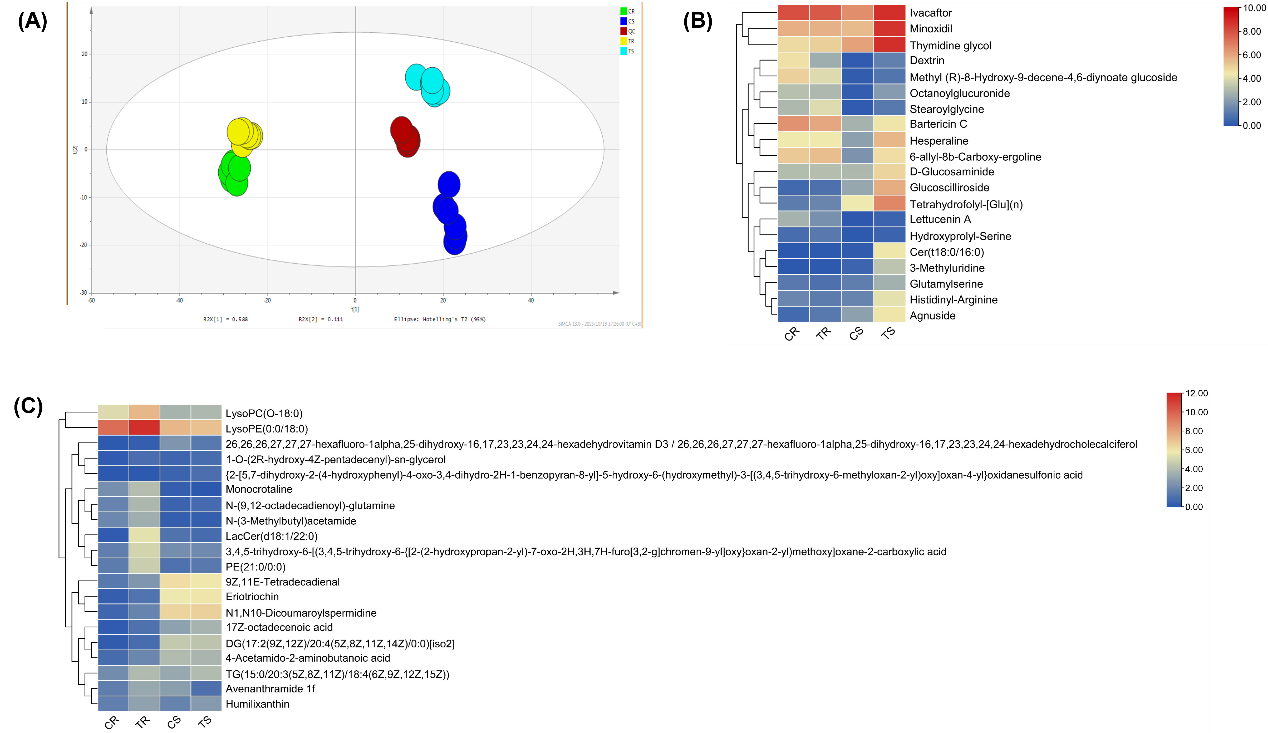


**Figure S2** Differentially accumulated metabolites (DAMs) among various treatments. Principal component analysis of DAMs in the roots and shoots of carbon nano sol (CNS)-treated tobacco seedlings (A). (B-C) Heat maps of the top 20 enriched DAMs in the roots (B) and shoots (C), respectively. C: mock; T: 0.3% CNS treatment. S: Shoot R: Root.


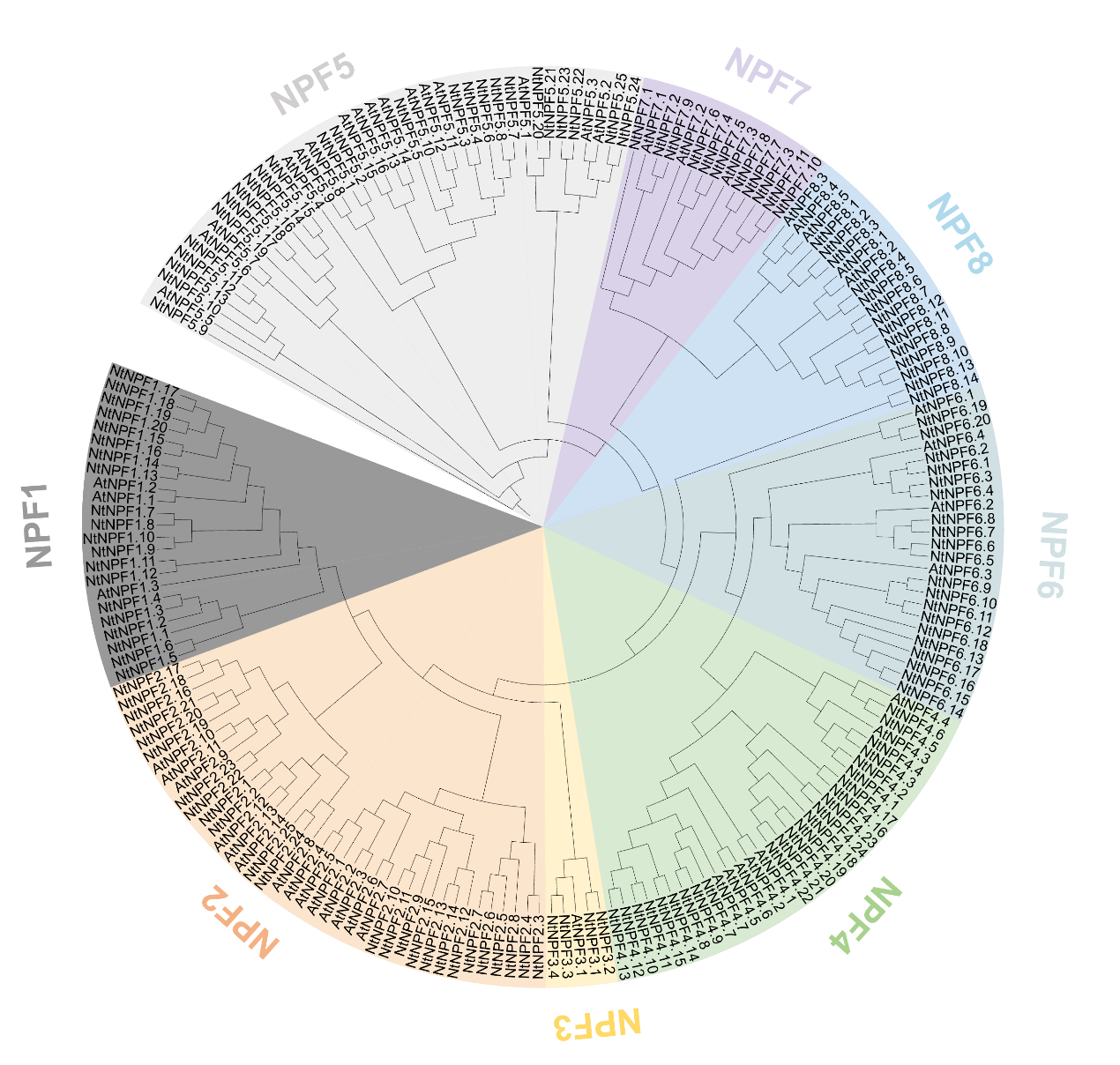


**Figure S3** Phylogenetic tree of the NPFs retrieved from tobacco and Arabidopsis. The phylogenetic tree was constructed according to the neighbor-joining method. The tree was generated using MEGA7.0 based on the NPF amino acid sequences retrieved from tobacco and Arabidopsis. The genes from each group are indicated by different colors. Rectangle size at the nodes represents the bootstrap values.


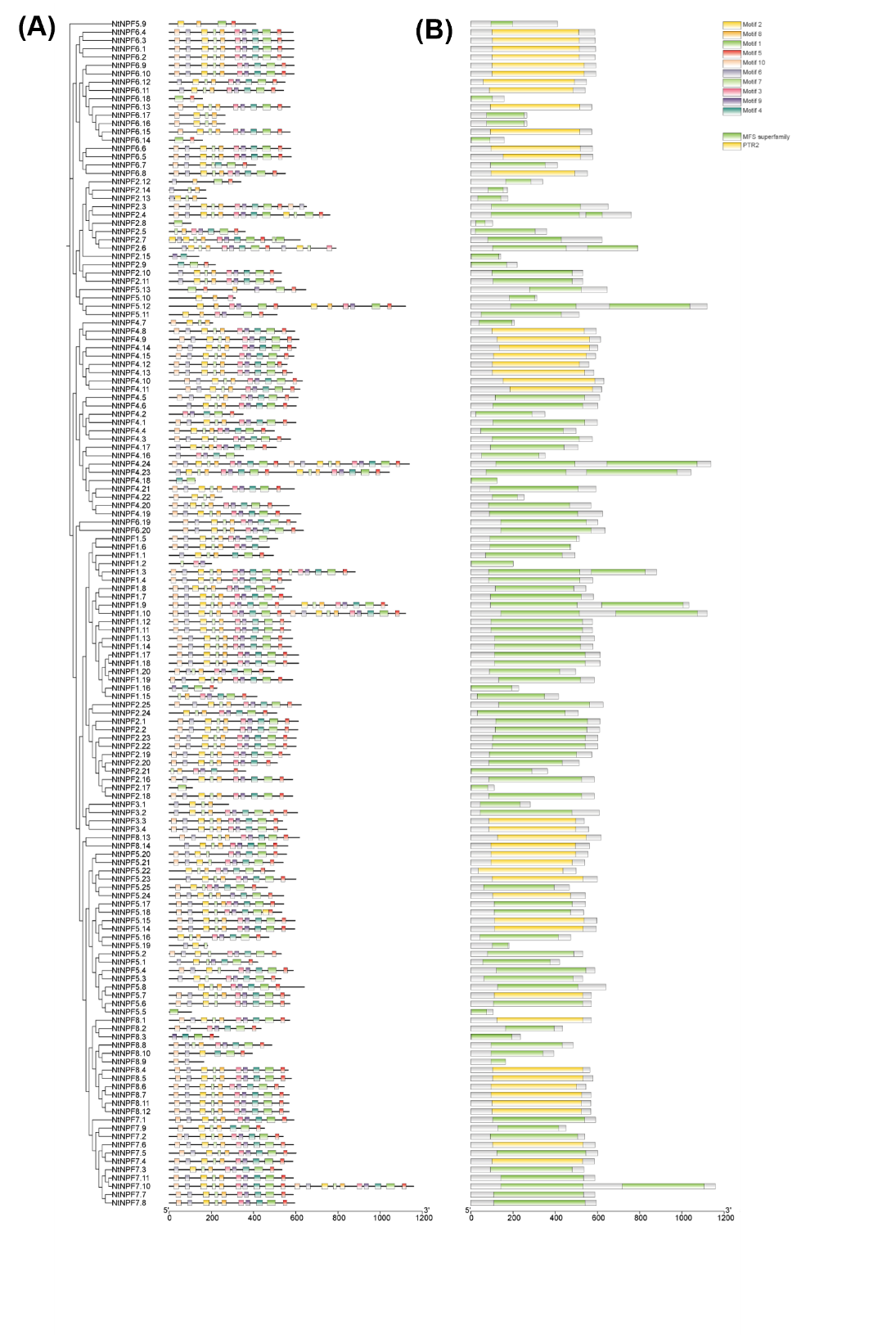


**Figure S4** Identification and characterization of the conserved motifs and domain in the NPF proteins in tobacco. (A) Motif composition of NPF proteins in tobacco. The colored boxes represent distinct conserved motifs (motifs 1–10), and the black lines represent NPF protein areas with no discovered motifs. (B) Domain analysis of the NPF family members.


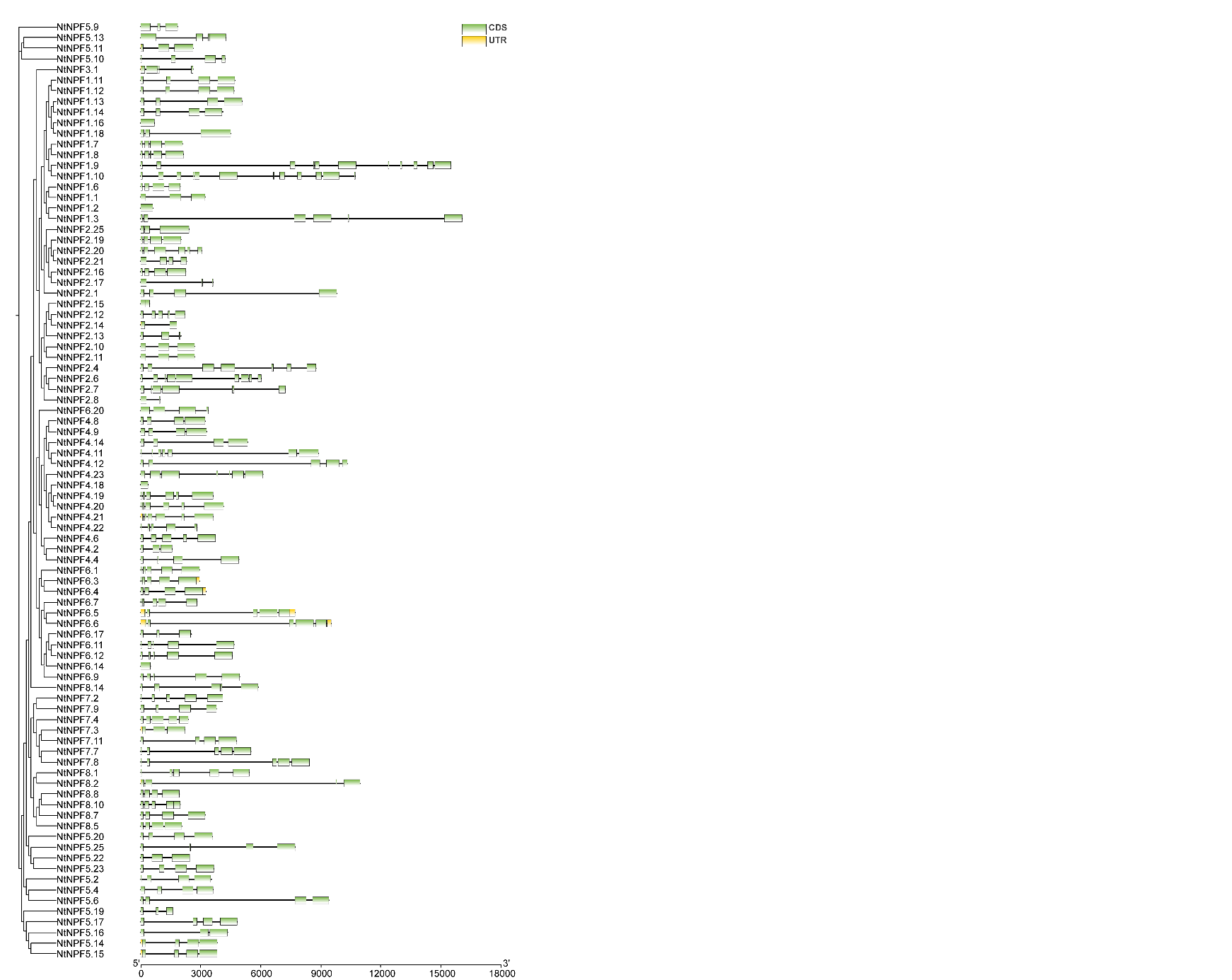


**Figure S5** Identification and characterization of the gene structure in the NPF proteins in tobacco. Exon-intron structures of the 88 genes in NPF gene families. Untranslated regions (UTRs) are shown by green boxes, while exons are shown by yellow boxes, and introns are shown by black lines.


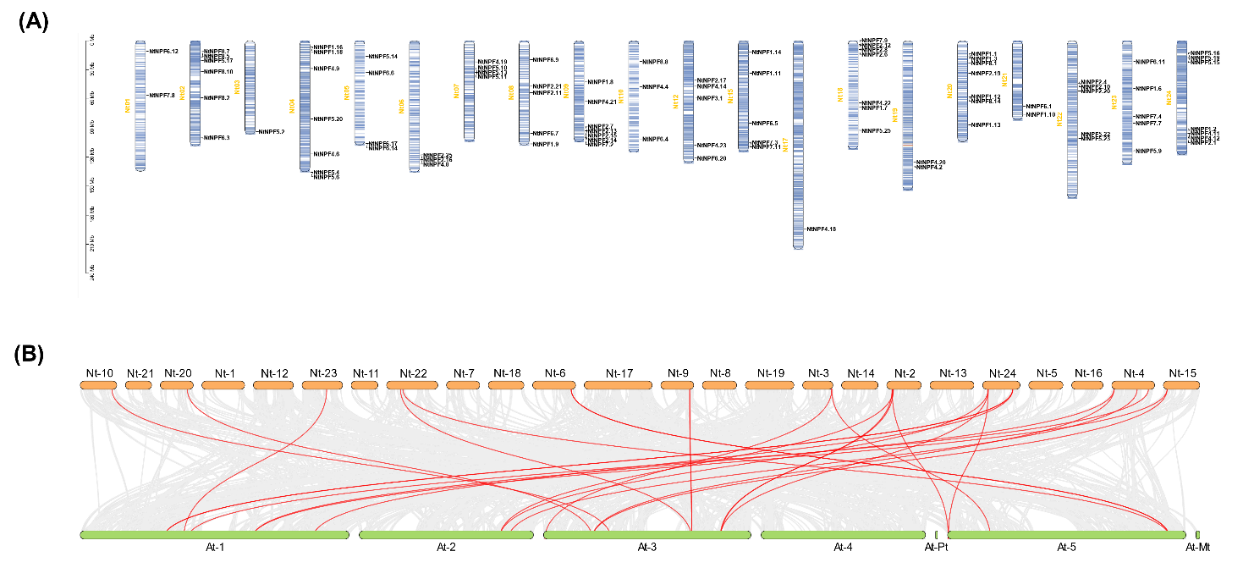


**Figure S6** Chromosomal location of the NPF family genes in tobacco and Synteny analysis of the NPF family genes between tobacco and Arabidopsis. Base pairs (bps) are represented by the scale. On the left side of each vertical bar, the chromosomal numbers are displayed. Gray lines indicate all collinear blocks within tobacco and Arabidopsis, while the red lines depict the orthologous relationships.


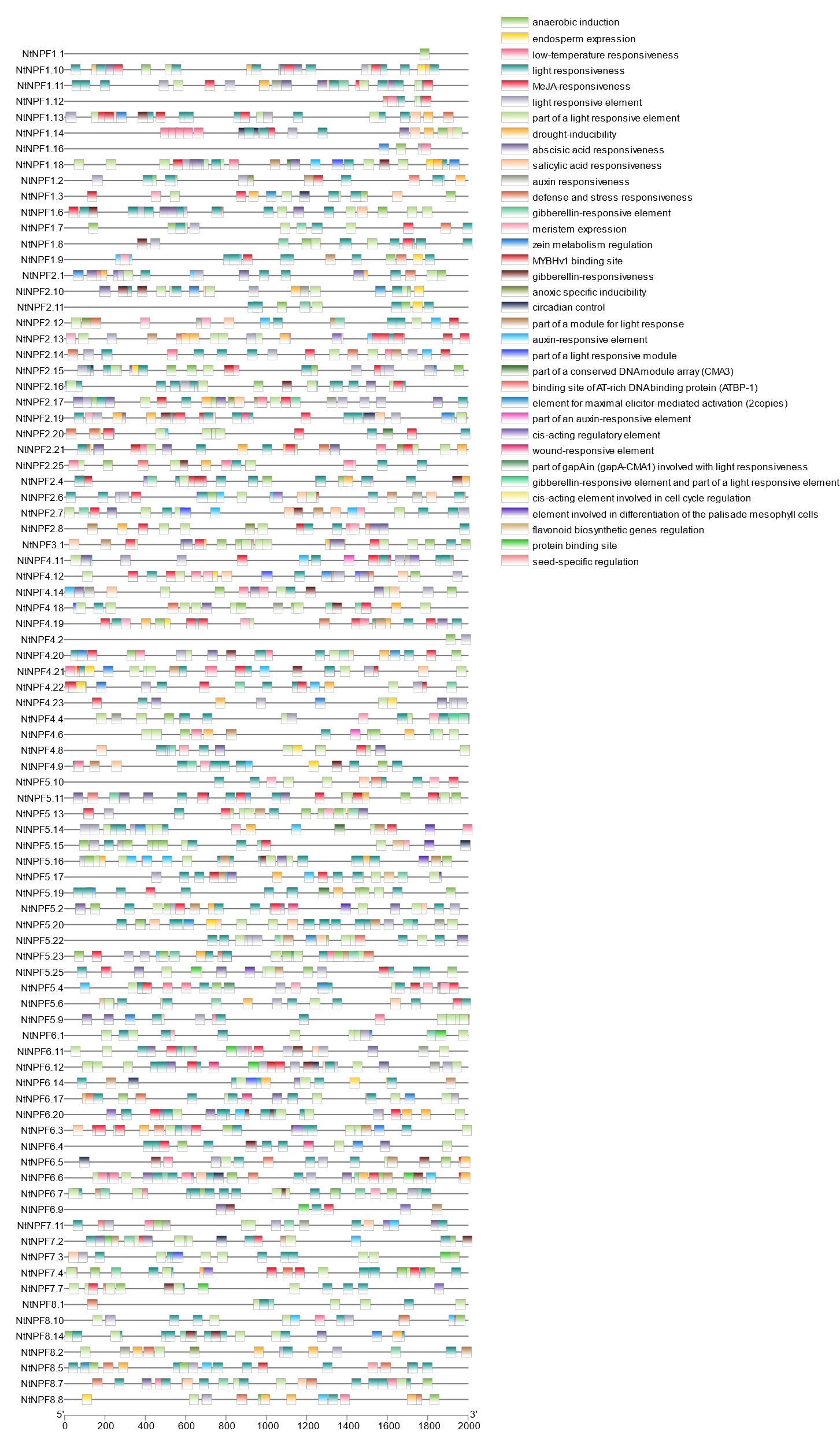


**Figure S7** Predicted *cis*-regulatory elements (CREs) in the promoter regions of *NtNPFs*. The *cis*-element identified by PlantCARE is based on the sequence of 2,000 bp upstream of the start codon of the 143 genes in *NPF* gene families.

**Figure S8** Differential expression profiling of genes involved in reactive oxygen species (ROS) production, scavenging, and phytohormone biosynthesis and the genes involved in the transport of phosphorus (P), magnesium (Mg), copper (Cu), boron (B), and potassium (K) in tobacco plants between the control and 0.3% carbon nano sol (CNS) treatment conditions. (A) Differential expression profiling of the genes involved in ROS production. RBOH, respiratory burst oxidase homologs. (B) General expression profiling of differentially expressed antioxidant enzyme genes. CAT, catalase; APX, ascorbate peroxidase; (C) Differential expression profiling of genes involved in auxin (IAA) biosynthesis. Tryptophan aminotransferases, YUCCA. (D) Differential expression profiling of genes involved in salicylic acid (SA) biosynthesis. phenylalanine ammonia lyase, PAL. (E) Differential expression profiling of genes involved in gibberellin (GA) biosynthesis. GAox, GA oxidase. (F) Differential expression profiling of genes involved in jasmonic acid (JA) biosynthesis. PLA1, phospholipase A1; AOS, allene oxide synthase; LOX, lipoxygenase. Differential expression profiling in the tobacco plants between the control and 0.3% CNS conditions. (G-J) Differential expression profiling of the genes involved in the transport of P (G), Mg (H), Cu (I), and K (J). PHT, phosphate transporter; MGT, magnesium transporter; COPT, copper transporter; HKT, high-affinity K^+^ transporter type; KCO, two-pore K^+^ channel; AKT, Arabidopsis K^+^ transporter. The heatmaps show gene expression levels as indicated by the FPKM values.

**Supplementary Table S1 Gene information of *NPF* in *N. tabacum***

| **Group** | **Gene name** | **Gene ID** |
| --- | --- | --- |
| NPF1 | *NtNPF1.1* | *Nitab4.5_0004963g0010* |
|  | *NtNPF1.2* | *Nitab4.5_0001474g0110* |
|  | *NtNPF1.3* | *Nitab4.5_0004963g0020* |
|  | *NtNPF1.4* | *Nitab4.5_0004033g0020* |
|  | *NtNPF1.5* | *Nitab4.5_0005197g0010* |
|  | *NtNPF1.6* | *Nitab4.5_0000151g0290* |
|  | *NtNPF1.7* | *Nitab4.5_0001734g0010* |
|  | *NtNPF1.8* | *Nitab4.5_0001139g0010* |
|  | *NtNPF1.9* | *Nitab4.5_0004391g0020* |
|  | *NtNPF1.10* | *Nitab4.5_0001004g0010* |
|  | *NtNPF1.11* | *Nitab4.5_0001309g0110* |
|  | *NtNPF1.12* | *Nitab4.5_0001737g0090* |
|  | *NtNPF1.13* | *Nitab4.5_0001968g0130* |
|  | *NtNPF1.14* | *Nitab4.5_0002859g0160* |
|  | *NtNPF1.15* | *Nitab4.5_0011683g0030* |
|  | *NtNPF1.16* | *Nitab4.5_0001315g0050* |
|  | *NtNPF1.17* | *Nitab4.5_0004564g0020* |
|  | *NtNPF1.18* | *Nitab4.5_0001315g0010* |
|  | *NtNPF1.19* | *Nitab4.5_0006926g0020* |
|  | *NtNPF1.20* | *Nitab4.5_0000671g0040* |
| NPF2 | *NtNPF2.1* | *Nitab4.5_0000679g0150* |
|  | *NtNPF2.2* | *Nitab4.5_0001055g0010* |
|  | *NtNPF2.3* | *Nitab4.5_0010939g0010* |
|  | *NtNPF2.4* | *Nitab4.5_0000270g0060* |
|  | *NtNPF2.5* | *Nitab4.5_0001698g0090* |
|  | *NtNPF2.6* | *Nitab4.5_0000533g0080* |
|  | *NtNPF2.7* | *Nitab4.5_0000888g0070* |
|  | *NtNPF2.8* | *Nitab4.5_0000533g0100* |
|  | *NtNPF2.9* | *Nitab4.5_0007582g0010* |
|  | *NtNPF2.10* | *Nitab4.5_0000270g0050* |
|  | *NtNPF2.11* | *Nitab4.5_0000372g0050* |
|  | *NtNPF2.12* | *Nitab4.5_0000533g0050* |
|  | *NtNPF2.13* | *Nitab4.5_0000888g0080* |
|  | *NtNPF2.14* | *Nitab4.5_0000888g0110* |
|  | *NtNPF2.15* | *Nitab4.5_0000888g0120* |
|  | *NtNPF2.16* | *Nitab4.5_0004048g0010* |
|  | *NtNPF2.17* | *Nitab4.5_0005357g0040* |
|  | *NtNPF2.18* | *Nitab4.5_0000642g0010* |
|  | *NtNPF2.19* | *Nitab4.5_0000983g0020* |
|  | *NtNPF2.20* | *Nitab4.5_0000003g0010* |
|  | *NtNPF2.21* | *Nitab4.5_0000909g0040* |
|  | *NtNPF2.22* | *Nitab4.5_0007642g0020* |
|  | *NtNPF2.23* | *Nitab4.5_0011731g0010* |
|  | *NtNPF2.24* | *Nitab4.5_0000642g0020* |
|  | *NtNPF2.25* | *Nitab4.5_0004048g0050* |
| NPF3 | *NtNPF3.1* | *Nitab4.5_0000133g0010* |
|  | *NtNPF3.2* | *Nitab4.5_0003333g0010* |
|  | *NtNPF3.3* | *Nitab4.5_0003330g0010* |
|  | *NtNPF3.4* | *Nitab4.5_0000874g0180* |
| NPF4 | *NtNPF4.1* | *Nitab4.5_0007689g0040* |
|  | *NtNPF4.2* | *Nitab4.5_0002261g0020* |
|  | *NtNPF4.3* | *Nitab4.5_0000460g0040* |
|  | *NtNPF4.4* | *Nitab4.5_0002159g0010* |
|  | *NtNPF4.5* | *Nitab4.5_0001710g0020* |
|  | *NtNPF4.6* | *Nitab4.5_0000079g0040* |
|  | *NtNPF4.7* | *Nitab4.5_0008153g0020* |
|  | *NtNPF4.8* | *Nitab4.5_0004605g0120* |
|  | *NtNPF4.9* | *Nitab4.5_0000785g0250* |
|  | *NtNPF4.10* | *Nitab4.5_0010420g0010* |
|  | *NtNPF4.11* | *Nitab4.5_0000679g0050* |
|  | *NtNPF4.12* | *Nitab4.5_0000679g0060* |
|  | *NtNPF4.13* | *Nitab4.5_0002969g0010* |
|  | *NtNPF4.14* | *Nitab4.5_0000417g0150* |
|  | *NtNPF4.15* | *Nitab4.5_0013559g0010* |
|  | *NtNPF4.16* | *Nitab4.5_0014978g0020* |
|  | *NtNPF4.17* | *Nitab4.5_0002507g0090* |
|  | *NtNPF4.18* | *Nitab4.5_0001225g0170* |
|  | *NtNPF4.19* | *Nitab4.5_0000541g0070* |
|  | *NtNPF4.20* | *Nitab4.5_0003511g0040* |
|  | *NtNPF4.21* | *Nitab4.5_0000081g0170* |
|  | *NtNPF4.22* | *Nitab4.5_0002303g0040* |
|  | *NtNPF4.23* | *Nitab4.5_0000068g0180* |
|  | *NtNPF4.24* | *Nitab4.5_0008935g0050* |
| NPF5 | *NtNPF5.1* | *Nitab4.5_0007185g0070* |
|  | *NtNPF5.2* | *Nitab4.5_0004905g0020* |
|  | *NtNPF5.3* | *Nitab4.5_0003329g0020* |
|  | *NtNPF5.4* | *Nitab4.5_0004233g0010* |
|  | *NtNPF5.5* | *Nitab4.5_0008515g0030* |
|  | *NtNPF5.6* | *Nitab4.5_0004233g0040* |
|  | *NtNPF5.7* | *Nitab4.5_0004154g0030* |
|  | *NtNPF5.8* | *Nitab4.5_0009715g0040* |
|  | *NtNPF5.9* | *Nitab4.5_0002651g0050* |
|  | *NtNPF5.10* | *Nitab4.5_0002650g0020* |
|  | *NtNPF5.11* | *Nitab4.5_0002650g0030* |
|  | *NtNPF5.12* | *Nitab4.5_0001264g0210* |
|  | *NtNPF5.13* | *Nitab4.5_0002650g0010* |
|  | *NtNPF5.14* | *Nitab4.5_0000828g0010* |
|  | *NtNPF5.15* | *Nitab4.5_0002170g0010* |
|  | *NtNPF5.16* | *Nitab4.5_0000652g0020* |
|  | *NtNPF5.17* | *Nitab4.5_0000362g0150* |
|  | *NtNPF5.18* | *Nitab4.5_0003084g0040* |
|  | *NtNPF5.19* | *Nitab4.5_0000652g0030* |
|  | *NtNPF5.20* | *Nitab4.5_0000263g0160* |
|  | *NtNPF5.21* | *Nitab4.5_0009443g0010* |
|  | *NtNPF5.22* | *Nitab4.5_0002389g0060* |
|  | *NtNPF5.23* | *Nitab4.5_0003295g0180* |
|  | *NtNPF5.24* | *Nitab4.5_0002008g0070* |
|  | *NtNPF5.25* | *Nitab4.5_0005658g0020* |
| NPF6 | *NtNPF6.1* | *Nitab4.5_0000622g0070* |
|  | *NtNPF6.2* | *Nitab4.5_0004644g0030* |
|  | *NtNPF6.3* | *Nitab4.5_0000482g0210* |
|  | *NtNPF6.4* | *Nitab4.5_0003449g0030* |
|  | *NtNPF6.5* | *Nitab4.5_0000763g0030* |
|  | *NtNPF6.6* | *Nitab4.5_0002116g0050* |
|  | *NtNPF6.7* | *Nitab4.5_0000163g0340* |
|  | *NtNPF6.8* | *Nitab4.5_0001750g0050* |
|  | *NtNPF6.9* | *Nitab4.5_0001292g0050* |
|  | *NtNPF6.10* | *Nitab4.5_0003531g0060* |
|  | *NtNPF6.11* | *Nitab4.5_0000312g0370* |
|  | *NtNPF6.12* | *Nitab4.5_0004430g0080* |
|  | *NtNPF6.13* | *Nitab4.5_0006367g0020* |
|  | *NtNPF6.14* | *Nitab4.5_0000712g0280* |
|  | *NtNPF6.15* | *Nitab4.5_0008372g0010* |
|  | *NtNPF6.16* | *Nitab4.5_0012102g0010* |
|  | *NtNPF6.17* | *Nitab4.5_0000712g0270* |
|  | *NtNPF6.18* | *Nitab4.5_0012102g0020* |
|  | *NtNPF6.19* | *Nitab4.5_0004349g0010* |
|  | *NtNPF6.20* | *Nitab4.5_0002569g0070* |
| NPF7 | *NtNPF7.1* | *Nitab4.5_0010616g0030* |
|  | *NtNPF7.2* | *Nitab4.5_0001146g0140* |
|  | *NtNPF7.9* | *Nitab4.5_0007607g0020* |
|  | *NtNPF7.4* | *Nitab4.5_0000166g0100* |
|  | *NtNPF7.5* | *Nitab4.5_0007712g0010* |
|  | *NtNPF7.6* | *Nitab4.5_0004264g0030* |
|  | *NtNPF7.7* | *Nitab4.5_0000018g0210* |
|  | *NtNPF7.8* | *Nitab4.5_0000764g0060* |
|  | *NtNPF7.3* | *Nitab4.5_0007026g0010* |
|  | *NtNPF7.10* | *Nitab4.5_0006398g0030* |
|  | *NtNPF7.11* | *Nitab4.5_0007026g0020* |
| NPF8 | *NtNPF8.1* | *Nitab4.5_0001881g0010* |
|  | *NtNPF8.2* | *Nitab4.5_0000356g0080* |
|  | *NtNPF8.3* | *Nitab4.5_0007317g0010* |
|  | *NtNPF8.4* | *Nitab4.5_0001837g0060* |
|  | *NtNPF8.5* | *Nitab4.5_0000008g0830* |
|  | *NtNPF8.6* | *Nitab4.5_0007563g0010* |
|  | *NtNPF8.7* | *Nitab4.5_0000008g0840* |
|  | *NtNPF8.8* | *Nitab4.5_0007908g0030* |
|  | *NtNPF8.9* | *Nitab4.5_0000261g0240* |
|  | *NtNPF8.10* | *Nitab4.5_0000490g0010* |
|  | *NtNPF8.11* | *Nitab4.5_0004718g0020* |
|  | *NtNPF8.12* | *Nitab4.5_0000744g0200* |
|  | *NtNPF8.13* | *Nitab4.5_0001091g0020* |
|  | *NtNPF8.14* | *Nitab4.5_0000577g0010* |

**Supplementary Table S2.** **One-to-one orthologous relationships of the NPF genes between *N. tabacum* and *A. thaliana*.**

| **Gene Name** | | **Gene ID** | |
| --- | --- | --- | --- |
| ***N. tabacum*** | ***A. thaliana*** | ***N. tabacum*** | ***A. thaliana*** |
| *NtNPF1.16*  *NtNPF1.14*  *NtNPF1.13*  *NtNPF1.16*  *NtNPF1.14*  *NtNPF2.25*  *NtNPF2.20*  *NtNPF2.1*  *NtNPF2.1*  *NtNPF2.14*  *NtNPF2.7*  *NtNPF2.4*  *NtNPF4.6*  *NtNPF4.11*  *NtNPF4.8*  *NtNPF5.20*  *NtNPF5.17*  *NtNPF5.16*  *NtNPF5.17*  *NtNPF5.16*  *NtNPF5.2*  *NtNPF5.2*  *NtNPF6.4*  *NtNPF7.4*  *NtNPF8.7*  *NtNPF8.7*  *NtNPF5.16* | AtNPF1.1  AtNPF1.1  AtNPF1.1  AtNPF1.2  AtNPF1.2  AtNPF2.11  AtNPF2.11  AtNPF2.12  AtNPF2.14  AtNPF2.6  AtNPF2.7  AtNPF2.7  AtNPF4.4  AtNPF4.5  AtNPF4.7  AtNPF5.1  AtNPF5.6  AtNPF5.6  AtNPF5.7  AtNPF5.7  AtNPF5.8  AtNPF5.9  AtNPF6.4  AtNPF7.3  AtNPF8.1  AtNPF8.2  AtNPF8.2 | Nitab4.5_0001315g0050.1  Nitab4.5_0002859g0160.1  Nitab4.5_0001968g0130.1  Nitab4.5_0001315g0050.1  Nitab4.5_0002859g0160.1  Nitab4.5_0004048g0050.1  Nitab4.5_0000003g0010.1  Nitab4.5_0000679g0150.1  Nitab4.5_0000679g0150.1  Nitab4.5_0000888g0110.1  Nitab4.5_0000888g0070.1  Nitab4.5_0000270g0060.1  Nitab4.5_0000079g0040.1  Nitab4.5_0000679g0050.1  Nitab4.5_0004605g0120.1  Nitab4.5_0000263g0160.1  Nitab4.5_0000362g0150.1  Nitab4.5_0000652g0020.1  Nitab4.5_0000362g0150.1  Nitab4.5_0000652g0020.1  Nitab4.5_0004905g0020.1  Nitab4.5_0004905g0020.1  Nitab4.5_0003449g0030.1  Nitab4.5_0000166g0100.1  Nitab4.5_0000008g0840.1  Nitab4.5_0000008g0840.1  Nitab4.5_0000652g0020.1 | AT3G16180.1  AT3G16180.1  AT3G16180.1  AT1G52190.1  AT1G52190.1  AT5G62680.1  AT5G62680.1  AT1G27080.1  AT1G69860.1  AT3G45660.1  AT3G45650.1  AT3G45650.1  AT1G33440.1  AT1G27040.1  AT5G62730.1  AT2G40460.1  AT2G37900.1  AT2G37900.1  AT3G53960.1  AT3G53960.1  AT5G14940.1  AT3G01350.1  AT3G21670.1  AT1G32450.1  AT3G54140.1  AT5G01180.1  AT5G01180.1 |

**Supplementary** **Table S3.** Type and number of cis-acting elements

| **Name** | **Numbers** |
| --- | --- |
| light responsive element | 945 |
| MeJA-responsiveness | 204 |
| abscisic acid responsiveness | 188 |
| anaerobic induction | 115 |
| gibberellin-responsive element | 82 |
| drought-inducibility | 66 |
| auxin responsiveness | 62 |
| low-temperature responsiveness | 54 |
| defense and stress responsiveness | 50 |
| salicylic acid responsiveness | 44 |
| MYBHv1 binding site | 31 |
| meristem expression | 29 |
| zein metabolism regulation | 28 |
| endosperm expression | 20 |
| circadian control | 14 |
| cis-acting regulatory element | 14 |
| element involved in differentiation of the palisade mesophyll cells | 11 |
| element for maximal elicitor-mediated activation (2copies) | 10 |
| protein binding site | 10 |
| anoxic specific inducibility | 9 |
